# Synthesis and glycosidation of building blocks of D-altrosamine

**DOI:** 10.3389/fchem.2022.945779

**Published:** 2022-09-26

**Authors:** Mariya Novakova, Anupama Das, Catherine Alex, Alexei V. Demchenko

**Affiliations:** ^1^ Department of Chemistry, Saint Louis University, St. Louis, MO, United States; ^2^ Department of Chemistry and Biochemistry, University of Missouri—St. Louis, One University Boulevard, St. Louis, MO, United States

**Keywords:** glycosylation, synthesis, sugars, glycan, oligosaccharides

## Abstract

Presented herein is a streamlined synthesis of building blocks of a rare sugar D-altrosamine. Also investigated was the glycosylation of different glycosyl acceptors with differentially protected altrosamine donors. High facial stereoselectivity was achieved with 3-*O*-picoloyl donors and reactive glycosyl acceptors *via* the H-bond-mediated aglycone delivery (HAD) pathway. In contrast, glycosidations of the altrosamine donor equipped with the 3-*O*-benzoyl group were poorly stereoselective.

## Graphical Abstract

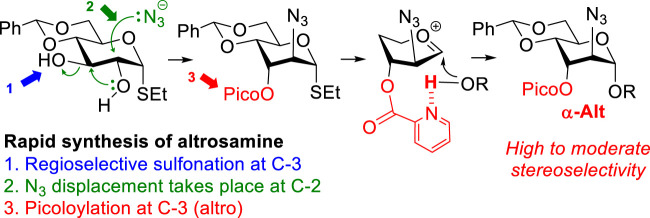

## Introduction

Due to significant progress in recent years, many glycans can now be obtained by using chemical methods and automated platforms (Panza et al., 2018). However, the availability of selectively protected sugar building blocks remains scarce, which hampers the scientific progress in the area of carbohydrate synthesis. Despite general improvements in the application of protecting group strategies in the mainstream carbohydrate research (Polyakova et al., 2015; Jager and Minnaard, 2016; Ágoston et al., 2016; Kulkarni et al., 2018; Volbeda et al., 2019; Wang and Demchenko, 2019), building blocks for the introduction of uncommon (rare or unnatural) sugars remain largely underdeveloped or not available at all (Emmadi and Kulkarni, 2014; Sanapala and Kulkarni, 2016a; Sanapala and Kulkarni, 2016b; Behera and Kulkarni, 2018; Wang et al., 2020).

Early reports for the synthesis of rare sugar D-altrose relied on the degradation of heptuloses (Ritchmyer et al., 1939) or modification of fructose (Araujo et al., 2012) among others. The synthesis of D-altrosamine could be achieved from 2,3-anhydroaltrose that was obtained from D-glucose precursors *via* a multi-step protocol (Vega-Perez et al., 1995; Nilsson and Norberg, 2000; Chiu et al., 2007; Shrestha et al., 2022). These building blocks have previously been used as synthons to access derivatized rare sugars. Nevertheless, altrosamine remains prohibitively expensive to be used as the starting material both for laboratory and industrial applications. Reported herein is a streamlined and scalable procedure for the synthesis of D-altrosamine building blocks. Also investigated was the first glycosidation of altrosamine donors with standard glycosyl acceptors.

## Results and discussion

**SCHEME 1:**
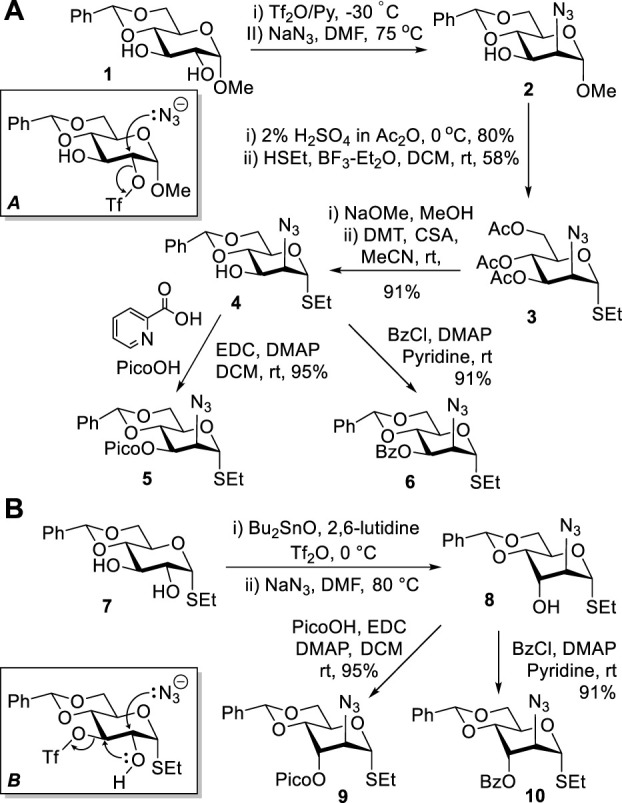
Previous synthesis of ManN_3_ building blocks **2–6** and the direct synthesis of D-altrosamine donors **9** and **10** from the D-gluco precursor **7**.

Previously, we developed methods to obtain mannosamine building blocks from methyl 4,6-*O*-benzylidene-α-D-glucopyranoside **1** (Alex et al., 2020a; Alex et al., 2020b). High regioselectivity of sulfonation of diol **1** with triflic anhydride at C-2 (Knapp et al., 1990) was the key to success in obtaining 2-azido-2-deoxy-d-mannopyranoside **2** (Scheme 1A). This reaction proceeded *via* a stereospecific nucleophilic displacement at C-2 with sodium azide in DMF (**A**) (Knapp et al., 1992). Compound **2** was then subjected to sequential acetolysis and a leaving group introduction to afford thioglycoside **3**. The latter was protected to afford 3-OH derivative **4**, which was then picoloylated to afford donor **5** or benzoylated to afford donor **6**. These donors were then used for stereoselective introduction of mannosides.

However, the synthesis of **5** and **6** remained somewhat tedious and required a lengthy and multi-step process to arrive at the desired compounds. In an effort to streamline the approach, we attempted to carry out the synthesis from thioglycoside **7** (Takeo et al., 1993) instead of the previously employed methyl glycoside **1**. All efforts to sulfonate thioglycoside **7** at position C-2 have failed, regardless of whether this reaction was performed in the presence of dibutyl tin oxide or not (Scheme 1B). Sulfonation was consistently directed to the C-3 position, and the subsequent nucleophilic displacement resulted in a cascade reaction with the anticipated pathway (**B**). Presumably, first, 2,3-cyclization would occur, and the resulting 2,3-epoxide (Walvoort et al., 2011) would then open upon the nucleophilic attack by N_3_ to afford D-altro-configured amino sugar **8**. This discovery led to a straightforward one-pot protocol for the synthesis of this rare sugar series and derivatives thereof. To elaborate upon this finding, we protected the 3-OH derivative **8** with picoloyl or benzoyl groups to afford glycosyl donors **9** and **10** in good yields of 95 and 91%, respectively (Scheme 1B).

We have previously reported that picolinyl or picoloyl (Pico) protecting groups at remote C-3, C-4, or C-6 positions of pyranose sugars can provide high facial *syn*-stereoselectivity in glycosylations (Yasomanee and Demchenko, 2012). This is due to the H-bond-mediated aglycone delivery (HAD) reaction pathway (Mannino et al., 2021). For the HAD reaction to take place, the Pico nitrogen of the glycosyl donor has to establish a hydrogen bond with the hydroxyl group of the glycosyl acceptor. Upon activation of the glycosyl donor, the glycosyl acceptor forms the glycosidic bond, which is *syn* with respect to the Pico substituent.

**SCHEME 2:**
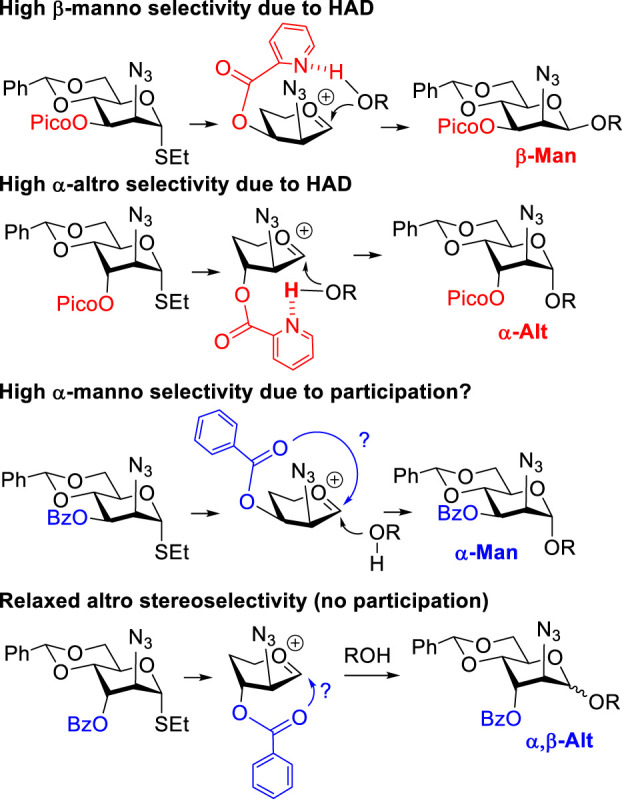
H-bond-mediated aglycone delivery and participation for mannosides and altrosides.

As previously proposed by our group (Alex et al., 2020a; Alex and Demchenko, 2021; Alex et al., 2021), the mannosamine donor equipped with the 3-O-Pico group will favor formation of the β-linked mannoside, as shown in Scheme 2. In the case of 3-O-benzoylated (3-Bz) mannosamine donor, excellent α-stereoselectivity was obtained. This result was explained by the occurrence of a remote participation of the 3-O-benzoyl group. Our expectations for glycosyl donors of the D-altrosamine series were opposed to those observed with D-manno donors. This is because of the orientation of the substituent at C-3. Thus, we anticipated that 3-Pico donors will provide preferential α-stereoselectivity whereas 3-Bz donor will be β-altro stereoselective due to the remote participation effect (Scheme 2).

We hypothesized that the remote 3-O-Pico group in donor **9** will act as an H-bond acceptor for the incoming nucleophile (hydroxyl group of the glycosyl acceptor). As a result, the formation of α-altrosides was anticipated. With that, we set up a series of glycosylations with common sugar acceptors **11**–**14** (Ranade et al., 2010), as shown in Table 1. When glycosyl donor **9** was coupled with 6-OH acceptor **11** in the presence of NIS/TfOH in 1,2-dichloroethane (1,2-DCE), the expected disaccharide **15** was obtained in 98% and complete α-altro stereoselectivity (entry 1). Since the HAD reaction pathway is absent in 3-Bz donor **10**, an opposing stereoselectivity was anticipated. However, the reaction between 3-Bz donor **10** and acceptor **11** yielded the corresponding disaccharide **16** in a high yield of 98%, albeit with modest stereoselectivity with α-anomer still favored (α/β = 3.0/1, entry 2). Both reactions were completed in 1 h. This result implies that the participation of the 3-Bz group seen for mannosides, (Crich et al., 2000) mannosamine glycosides, (Alex et al., 2020a; Alex et al., 2020b; Alex and Demchenko, 2021; Alex et al., 2021; Alex and Demchenko, 2022), and other sugar series, (Ustyuzhanina et al., 2006; Baek et al., 2009; Komarova et al., 2013; Komarova et al., 2014; Komarova et al., 2016) is practically ineffective in case of D-altro-configured sugars.

**TABLE 1:** Glycosidation of donors **9** and **10** with glycosyl acceptors **11–14**.

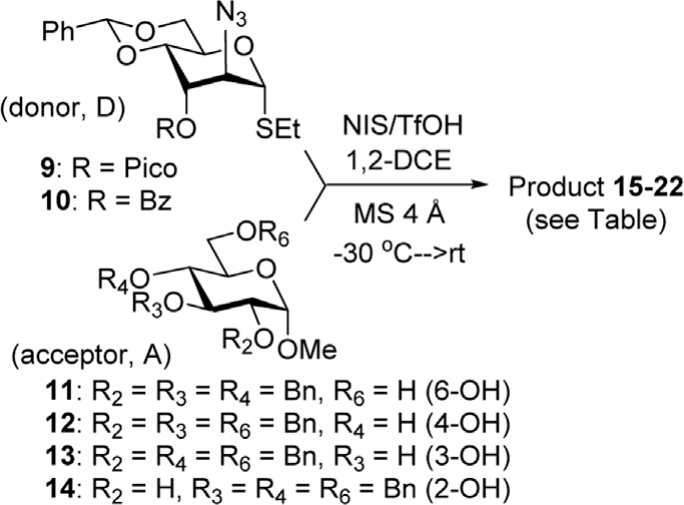		
Entry	D + A	Product: yield, stereoselectivity
1	**9 + 11**	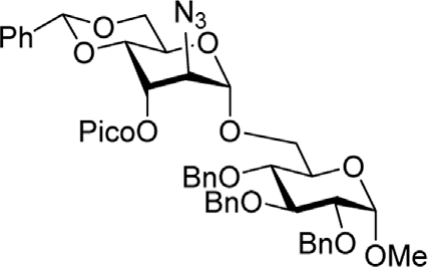
		**15**: 98%, α/β > 25/1
2	**10 + 11**	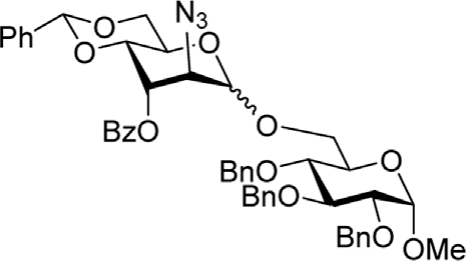
		**16**: 98%, α/β = 3.0/1
3	**9 + 12**	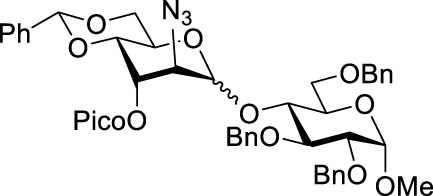
		**17**: 71%, α/β = 1.7/1
4	**10 + 12**	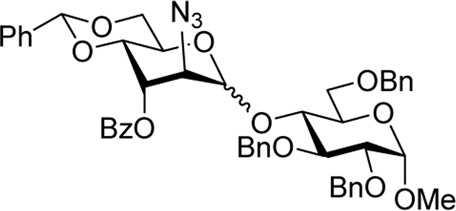
		**18**: 84%, α/β = 1.0/1
5	**9 + 13**	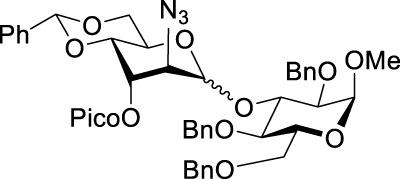
		**19**: 63%, α/β = 2.2/1
6	**10 + 13**	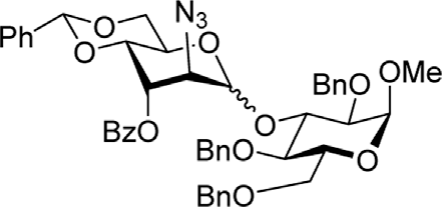
		**20**: 80%, α/β = 2.5/1
7	**9 + 14**	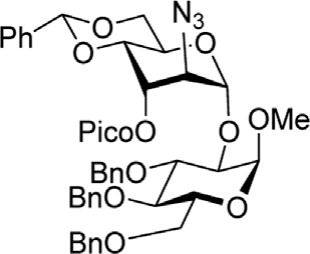
		**21**: 65%, α/β > 25/1
8	**10** + **14**	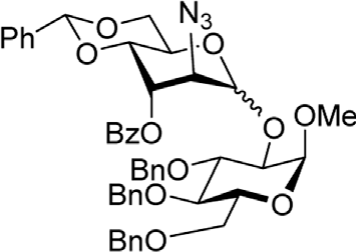
		**22**: 66%, α/β = 1.5/1

When the reaction of 4-OH glycosyl acceptor **12** was conducted with glycosyl donor **9** under the promotion of NIS/TfOH, disaccharide **17** was obtained in 71% yield, albeit with poor stereoselectivity (α/β = 1.7/1, entry 3). When 3-Bz donor **10** was glycosidated with acceptor **12**, disaccharide **18** was obtained in 84% yield with no stereoselectivity (α/β = 1.0/1, entry 4). A very similar trend was achieved in the reaction of 3-OH acceptor **13** with glycosyl donors **9** and **10**. The corresponding disaccharides **19** and **20** were achieved in good yields of 63–80% but with modest stereoselectivity in both cases (α/β = 2.2–2.5/1, entries 5–6). When glycosyl donor **9** was glycosidated with 2-OH acceptor **14**, disaccharide **21** was isolated in a good yield of 65% and with complete α-altro selectivity (entry 7). The reaction between 3-Bz glycosyl donor **10** and glycosyl acceptor **14** produced the corresponding disaccharide **22** in 66% yield, albeit with poor stereoselectivity (α/β = 1.5/1, entry 2). All glycosylations of secondary acceptors **12–14** were completed in 2 h. These results confirm the general trend previously seen in some HAD reactions wherein poorly nucleophilic acceptors provided lower stereoselectivity.

**SCHEME 3:**
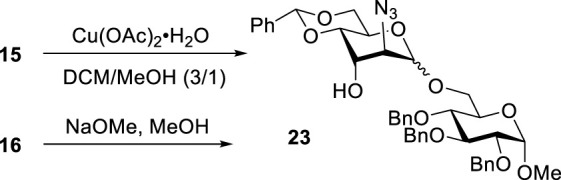
Deprotection of 15 and 16 to understand stereoselectivity.

To confirm the stereoselectivity observed, we removed 3-ester groups from disaccharides **15** and **16** (Scheme 3). The comparison of the NMR data on the resulting disaccharide **23** ultimately confirmed the preferential α-altrosamine configuration of disaccharides obtained with 3-Bz donor **10**.

In conclusion, a useful method for the synthesis of building blocks of D-altrosamine is reported. Also investigated was a stereoselective synthesis of α-glycosides of altrosamine. High facial stereoselectivity achieved with 3-*O*-picoloyl donors with reactive (6-OH and 2-OH) glycosyl acceptors was explained by the occurrence of the HAD reaction pathway. Stereoselectivity of reactions with 3-OH and 4-OH glycosyl acceptors was low because the HAD reaction was known to be less effective with poorly reactive acceptors (Yasomanee and Demchenko, 2012). Glycosidations of altrosamine donors equipped with the 3-*O*-benzoyl group were poorly stereoselective. Further investigation of these reactions is currently underway in our laboratory.

## Experiment

In general, the reactions were performed using commercial reagents. The ACS grade solvents used for reactions were purified and dried in accordance with the standard procedures. Column chromatography was performed on silica gel 60 (70–230 mesh), and reactions were monitored by TLC on Kiesel gel 60 F254. The compounds were detected by examination under UV light and by charring with 10% sulfuric acid in methanol. The solvents were removed under reduced pressure at <40°C. ClCH_2_.CH_2_Cl (1,2-DCE) was distilled from CaH_2_ directly prior to application. Anhydrous DMF was used. Molecular sieves (4 Å), used for reactions, were crushed and activated in *vacuo* at 390°C during 8 h in the first instance and then for 2–3 h at 390°C directly prior to application. Optical rotations were measured by using a ‘*J*asco P-2000’ polarimeter. ^1^H NMR spectra were recorded in CDCl_3_ at 400 MHz, and ^13^C NMR spectra were recorded in CDCl_3_ at 100 or 175 MHz. The ^1^H NMR chemical shifts are referenced to tetramethyl silane (TMS, δ_H_ = 0 ppm) or CDCl_3_ (δ_H_ = 7.26 ppm) for ^1^H NMR spectra for solutions in CDCl_3_. The ^13^C NMR chemical shifts are referenced to the central signal of CDCl_3_ (δ_C_ = 77.00 ppm) for solutions in CDCl_3_. Compound purity or compound ratios were accessed or calculated by comparing the integration intensities of the relevant signals in their ^1^H NMR spectra. Accurate mass spectrometry determinations were performed using the Agilent 6230 ESI-TOF LC/MS mass spectrometer.

## Synthesis of glycosyl donors 9 and 10

Ethyl 2-azido-4,6-O-benzylidene-2-deoxy-1-thio-α-D-altropyranoside **(8):** lutidine (4.80 ml, 41.6 mmol), and Bu_2_SnO (0.10 g, 0.41 mmol) were added to a stirring solution of ethyl 4,6-*O*-benzylidene-1-thio-α-D-glucopyranoside (**7**, (Takeo et al., 1993) 2.60 g, 8.32 mmol) in CH_2_Cl_2_ (30 ml) under argon at room temperature. The resulting mixture was cooled to 0°C; Tf_2_O (4.90 ml, 29.1 mmol) was added dropwise. The mixture was then stirred for 1 h at 0°C. The reaction mixture was then diluted with CH_2_Cl_2_ (250 ml) and washed with H_2_O (5 × 50 ml). The organic phase was separated, dried with Na_2_SO_4_, concentrated under reduced pressure, and dried in *vacuo*. The crude residue was dissolved in DMF (50 ml), NaN_3_ (2.70 g, 41.6 mmol) was added, and the resulting mixture was stirred under argon for 16 h at 80°C. The reaction mixture was then diluted with CH_2_Cl_2_ (250 ml) and washed with H_2_O (5 × 50 ml). The organic phase was separated, dried with Na_2_SO_4_, and concentrated under reduced pressure. The residue was purified by column chromatography on silica gel (ethyl acetate–hexane gradient elution) to afford the title compound as a white amorphous solid. Analytical data for **8**: R_*f*_ = 0.50 (ethyl acetate/hexane, 3/7, v/v); [α]_D_^24^ +95.6 (*c* 1.0, CHCl_3_); ^1^H NMR (400 MHz, CDCl_3_): δ 7.52–7.35 (m, 5H, aromatic), 5.65 (s, 1H, >C*H*Ph), 5.23 (s, 1H, H-1), 4.62 (m, 1H, *J*_5,6a_ = 5.1, *J*_5,6b_ = 10.1 Hz, H-5), 4.29 (dd, 1H, *J*_6a,6b_ = 10.3 Hz, H-6a), 4.13 (dd, 1H, *J*_3,4_ = 2.8 Hz, H-3), 4.00 (d, 1H *J*_2,3_ = 3.2 Hz, H-2), 3.91 (dd, 1H, *J*_4,5_ = 9.8 Hz, H-4), 3.81 (m, 1H, H-6b), 2.88 (d, 1H, OH), 2.72–2.61 (m, 2H, SCH_2_), and 1.30 (t, 3H, SCH_2_C*H*_3_) ppm; ^13^C NMR (100 MHz, CDCl_3_): δ 137.0, 129.4, 128.4 (x2), 126.2 (x2), 102.3, 82.4, 76.3, 68.9, 67.6, 64.0, 58.8, 27.1, and 14.9 ppm; and HRMS [M + Na]^+^ calcd for C_15_H_19_N_3_O_4_SNa 360.0994; found 360.0995.

Ethyl 2-azido-4,6-O-benzylidene-2-deoxy-3-O-picoloyl-1-thio-α-D-altropyranoside **(9)**: picolinic acid (0.39 g, 3.2 mmol), EDC (0.61 g, 3.2 mmol), and DMAP (51 mg, 0.42 mmol) were added to a solution of compound **8** (0.71 g, 2.1 mmol) in dry CH_2_Cl_2_ (10 ml), and the resulting mixture was stirred under argon for 3 h at room temperature. After that, the reaction mixture was diluted with CH_2_Cl_2_ (20 ml) and washed with water (10 ml), sat. aq. NaHCO_3_ (10 ml), and water (2 × 10 ml). The organic phase was separated, dried with Na_2_SO_4_, and concentrated under reduced pressure. The residue was purified by column chromatography on silica gel (ethyl acetate–hexane gradient elution) to afford the title compound as a white amorphous solid in 95% yield (1.32 g, 2.98 mmol). Analytical data for **9**: R_*f*_ = 0.50 (ethyl acetate/hexane, 1/1, v/v); [α]_D_^24^ + 57.4 (*c =* 1.0, CHCl_3_); ^1^H NMR (400 MHz, CDCl_3_): δ 8.82 (dd, 1H, aromatic), 8.36 (d, 1H, *J* = 7.8 Hz, aromatic), 7.88 (m, 1H, aromatic), 7.30–7.55 (m, 6H, aromatic), 5.64 (s, 1H, >C*H*Ph), 5.59 (dd, 1H, *J*_3,4_ = 3.2 Hz, H-3), 5.30 (s, 1H, H-1), 4.75 (m, 1H, *J*_5,6a_ = 5.2, *J*_5,6b_ = 10.1 Hz, H-5), 4.33 (dd, 1H, *J*_6a,6b_ = 10.5 Hz, H-6a), 4.22 (d, 1H, *J*_2,3_ = 4.0 Hz, H-2), 4.14 (dd, 1H, *J*_4,5_ = 9.8 Hz, H-4), 3.86 (dd, 1H, H-6b), 2.69 (m, 2H, SCH_2_), and 1.31 (t, 3H, SCH_2_C*H*_3_) ppm; ^13^C NMR (100 MHz, CDCl_3_): δ 163.3, 150.4, 147.2, 137.1, 136.9, 129.1, 128.3 (x2), 127.1, 126.1 (x2), 125.9, 102.1, 82.5, 74.3, 68.9, 68.5, 62.4, 60.0, 27.2, and 15.1 ppm; and HRMS [M + H]^+^ calcd for C_21_H_23_N_4_O_5_S 443.1395; found 443.1384.

Ethyl 2-azido-3-O-benzoyl-4,6-O-benzylidene-2-deoxy-1-thio-α-D-altropyranoside **(10)**: benzoyl chloride (0.25 ml, 2.0 mmol) and DMAP (24 mg, 0.20 mmol) were added to a solution of compound **8** (0.33 g, 1.0 mmol) in pyridine (15 ml), and the resulting mixture was stirred under argon for 2 h at room temperature. After that, the reaction was quenched with MeOH (5 ml), the volatiles were removed under reduced pressure, and the residue was co-evaporated with toluene. The resulting residue was diluted with CH_2_Cl_2_ (20 ml) and washed with water (10 ml), 1 N aq. HCl (10 ml), and water (2 × 10 ml). The organic phase was separated, dried with Na_2_SO_4_, and concentrated under reduced pressure. The residue was purified by column chromatography on silica gel (ethyl acetate–hexane gradient elution) to give the title compound as an off-white amorphous solid in 91% yield (0.40 g, 0.91 mmol). Analytical data for **10**: R_*f*_ = 0.50 (ethyl acetate/hexane, 2/3, v/v); [α]_D_^24^ +68.6 (*c* 1.0, CHCl_3_); ^1^H NMR (700 MHz, CDCl_3_): δ 8.28–8.25 (m, 2H, aromatic), 8.18–8.16 (m, 1H, aromatic), 7.66–7.61 (m, 1H, aromatic), 7.54–7.50 (m, 2H, aromatic), 7.46–7.42 (m, 2H, aromatic), 7.35–7.32 (m, 2H, aromatic), 5.67 (s, 1H, >C*H*Ph), 5.56 (dd, 1H, *J*_3,4_ = 3.2 Hz, H-3), 5.34 (s, 1H, H-1), 4.83 (m, 1H, *J*_5,6a_ = 5.2, *J*_5,6b_ = 10.1 Hz, H-5), 4.38 (dd, 1H, *J*_6a,6b_ = 10.5 Hz, H-6a), 4.26 (d, 1H, *J*_2,3_ = 3.6 Hz, H-2), 4.17 (dd, 1H, *J*_4,5_ = 9.8 Hz, H-4), 3.90 (dd, 1H, H-6b), 2.77–2.67 (m, 2H, SCH_2_), and 1.35 (t, 3H, SCH_2_C*H*_3_) ppm; ^13^C NMR (100 MHz, CDCl_3_): δ 165.5, 137.0, 133.7, 133.4, 130.2 (×2), 129.3, 129.1, 128.5 (x2), 128.2, 126.1 (×2), 102.2, 82.5, 74.4, 68.9, 67.9, 62.4, 60.0, 27.2, and 15.1 ppm; and HRMS [M + H]^+^ calcd for C_22_H_24_N_3_O_5_S 442.1436; found 442.1431.

## Synthesis of disaccharides 15–22

General procedure for glycosylation in the presence of NIS/TfOH: a mixture of glycosyl donor (0.1 mmol), glycosyl acceptor (0.09 mmol), and freshly activated molecular sieves (4 Å, 100 mg) in 1,2-DCE (2.0 ml) was stirred under argon for 1 h at room temperature. The mixture was then cooled to −30°C, *N*-iodosuccinimide (NIS, 0.22 mmol) and trifluoromethanesulfonic acid (TfOH, 2.0 µl, 0.02 mmol) were added, and the resulting mixture was allowed to warm to ambient temperature and stirred for 1–2 h at room temperature. After that, the solids were filtered off and washed successively with CH_2_Cl_2_. The combined filtrate (30–40 ml) was washed with water (10 ml), 10% sodium thiosulfate (Na_2_S_2_O_3_, 10 ml), and water (2 × 10 ml). The organic phase was separated, dried with Na_2_SO_4_, and concentrated under reduced pressure. The residue was purified by column chromatography on silica gel (acetone–toluene gradient elution) to afford corresponding disaccharide derivatives. Anomeric ratios (or anomeric purity) were determined by the comparison of the integral intensities of the relevant signals in ^1^H NMR spectra.

Methyl 6-O-(2-azido-4,6-O-benzylidene-2-deoxy-3-O-picoloyl-α-D-altropyranosyl)-2,3,4-tri-O-benzyl-α-D-glucopyranoside **(15)**: the title compound was obtained as an off-white amorphous solid from glycosyl donor **9** and acceptor **11** ^29^ in 98% yield. Analytical data for **15**: R_*f*_ = 0.40 (ethyl acetate/toluene, 3/7, v/v); [α]_D_^24^ +42.5 (*c* 1.0, CHCl_3_); ^1^H NMR (400 MHz, CDCl_3_): δ 8.64 (dd, 1H, aromatic), 8.04 (dd, 1H, aromatic), 7.50–7.00 (m, 22H, aromatic), 5.61 (s, 1H, >C*H*Ph), 5.51 (dd, 1H, *J*_3’,4’_ = 3.0 Hz, H-3′), 4.95 (d, 1H, C*H*Ph), 4.92 (s, 1H, H-1′), 4.69–4.84 (m, 3H, 3 x C*H*Ph), 4.58 (d, 1H, C*H*Ph), 4.46 (m, 2H, H-5′, C*H*Ph), 4.45 (d, 1H, *J*_1,2_ = 3.5 Hz, H-1), 4.23 (d, 1H, *J*_2′,3′_ = 2.8 Hz, H-2′) 4.20 (dd, 1H, *J*_6’a,6’b_ = 10.4 Hz, H-6′a), 4.09 (dd, 1H, *J*_4’,5’_ = 9.7 Hz, H-4′), 4.01 (dd, 1H, *J*_6a,6b_ = 11.4 Hz, H-6a), 3.91 (dd, 1H, *J*_3,4_ = 9.3 Hz, H-3), 3.77 (dd, 1H, H-6b′), 3.71–3.67 (m, 1H, H-5), 3.63 (dd, 1H, H-6b), 3.48 (dd, 1H, *J*_4,5_ = 9.5 Hz, H-4), 3.27 (dd, 1H, *J*_2,3_ = 9.3 Hz, H-2), and 3.22 (s, 3H, CH_3_) ppm; ^13^C NMR (100 MHz, CDCl_3_): δ 164.2, 150.4, 147.4, 138.7, 138.2, 138.1, 137.0, 136.9, 129.1, 128.5 (x2), 128.4 (x2), 128.3 (x2), 128.2 (x4), 128.0 (x2), 127.9, 127.7 (x2), 127.5 (x2), 126.9, 126.1 (x2), 125.2, 102.1, 98.9, 98.0, 81.7, 80.1, 75.7, 74.8, 73.8, 73.4 (x2), 69.8, 69.0, 68.9, 67.2, 60.1, 59.3, and 55.2 ppm; and HRMS [M + Na]^+^ calcd for C_47_H_48_N_4_O_11_Na 867.3238; found 867.3212.

Methyl 6-O-(2-azido-3-O-benzoyl-4,6-O-benzylidene-2-deoxy-D-altropyranosyl)-2,3,4-tri-O-benzyl-α-D-glucopyranoside **(16)**: the title compound was obtained as an off-white sticky semi-solid from glycosyl donor **10** and acceptor **11** ^29^ in 98% yield (α/β = 3.0/1). Selected analytical data for α-**16**: R_*f*_ = 0.40 (ethyl acetate/toluene, 3/7, v/v); ^1^H NMR (400 MHz, CDCl_3_): δ 8.11–7.98 (m, 3H, aromatic), 7.49 (m, 1H, aromatic), 7.43–7.09 (m, 21H, aromatic), 5.62 (s, 1H, >C*H*Ph), 5.39 (dd, 1H, *J*_3’,4’_ = 3.1 Hz, H-3′), 4.91 (s, 1H, H-1′), 4.76–4.71 (m, 4H, H-5′, 3 x C*H*Ph), 4.66–4.54 (m, 4H, H-6′a, 3 x C*H*Ph), 4.41 (d, 1H, *J*_1,2_ = 3.5 Hz, H-1), 4.24 (d, 1H, *J*_2,3_ = 3.1 Hz, H-2′), 4.07 (dd, 1H, *J*_4’,5’_ = 9.7 Hz, H-4′), 3.90 (dd, 1H, *J*_3,4_ = 9.2 Hz, H-3), 3.79 (m, 2H, H-6′b), 3.71–3.63 (m, 2H, H-5, 6 b), 3.36–3.27 (m, 2H, H-2, 4), and 3.14 (s, 3H, CH_3_) ppm; ^13^C NMR (100 MHz, CDCl_3_): δ 165.9, 165.1, 138.7 (×2), 138.3, 138.1 (×2), 138.0, 137.1, 136.9, 133.7, 133.3, 129.8 (×2), 129.7, 129.6 (×2), 129.4, 129.2, 129.1, 129.0, 128.7, 128.6 (×2), 128.5 (×5), 128.4 (×4), 128.3 (×4), 128.2, 128.1 (×3), 128.0 (×6), 127.9 (×2), 127.8, 127.7, 127.6 (×6), 126.1 (×5), 102.3, 102.1, 99.1 (x2), 98.0, 97.9, 82.1, 81.7, 80.1, 79.8, 77.6, 77.1, 75.7, 74.9, 74.7, 74.0, 73.9, 73.5 (x2), 70.1, 69.7, 69.6, 69.1, 68.9, 68.7, 68.5 (x2), 67.3, 64.7, 61.0, 60.1, 59.4, 55.2, and 55.1 ppm; and HRMS [M + H]^+^ calcd for C_48_H_50_O_11_N_3_ 844.3443; found 844.3440.

Methyl 4-O-(2-azido-4,6-O-benzylidene-2-deoxy-3-O-picoloyl-D-altropyranosyl)-2,3,6-tri-O-benzyl-α-D-glucopyranoside **(17)**: the title compound was obtained as a white amorphous solid from glycosyl donor **9** and acceptor **12** ^29^ in 71% yield (α/β = 1.7/1). Selected analytical data for α-**17**: R_*f*_ = 0.40 (ethyl acetate/toluene, 3/7, v/v); ^1^H NMR (400 MHz, CDCl_3_): δ 8.77 (dd, 1H, aromatic), 8.07 (dd, 1H, aromatic), 7.73 (dd, 1H, aromatic), 7.47–7.23 (m, 21H, aromatic), 5.60 (s, 1H, >CHPh), 5.45 (dd, 1H, *J*_3’,4’_ = 3.0 Hz, H-3′), 5.20 (s, 1H, H-1′), 5.00 (d, IH, C*H*Ph), 4.70 (d, 1H, C*H*Ph), 4.65–4.54 (m, 3H, C*H*Ph), 4.57 (d, 1H, *J*_1,2_ = 3.0 Hz, H-1) 4.50 (d, 1H, C*H*Ph), 4.46–4.40 (m, 1H, *J*_5’,6a’_ = 10.1, *J*_5’,6b’_ = 5.2 Hz, H-5′), 4.17 (dd, 1H, *J* = 10.6 Hz, H-6a′), 4.08 (dd, 1H, *J*_4’,5’_ = 9.8 Hz, H-4′), 3.91 (d, 1H, *J*_2’,3’_ = 3.0 Hz, H-2′), 3.85 (dd, 1H, *J*_6a,6b_ = 10.7 Hz, H-6a), 3.78–3.69 (m, 4H, H-3, 4, 6b, 6 b’), 3.57 (m, 1H, *J*_5,6a_ = 5.4 Hz, H-5), 3.52 (dd, 1H, *J*_2,3_ = 9.4 Hz, H-2), and 3.33 (s, 3H, CH_3_) ppm; ^13^C NMR (100 MHz, CDCl_3_): δ 163.8, 150.3, 149.8, 147.7, 138.2, 138.1, 138.0, 137.9, 137.8, 137.7, 137.1 (×2), 137.0, 136.8, 129.2, 129.1, 128.6 (×4), 128.5 (×3), 128.4 (×5), 128.3, 128.2 (×7), 128.1 (×2), 127.9, 127.8 (×2), 127.7 (×5), 127.6 (×5), 127.0 (×2), 126.3, 126.2, 126.1 (×4), 126.0, 125.3, 125.2, 102.2, 102.1, 99.8, 99.7, 97.8 (×2), 81.5 (×2), 80.4, 80.3, 80.2, 76.5, 75.9, 75.4, 73.9, 73.7, 73.6, 73.2 (×2), 69.8, 69.3, 69.1, 69.0 (×2), 68.9, 68.7, 67.5, 61.7, 60.0, 59.9, 58.9, 55.3, 55.2, and 52.9 ppm; and HRMS [M + Na]^+^ calcd for C_47_H_48_N_4_O_11_Na 867.3238; found 867.3212.

Methyl 4-O-(2-azido-3-O-benzoyl-4,6-O-benzylidene-2-deoxy-D-altropyranosyl)-2,3,6-tri-O-benzyl-α-D-glucopyranoside **(18)**: the title compound was obtained as a white amorphous solid from glycosyl donor **10** and acceptor **12** in 84% yield (α/β = 1.0/1). Selected analytical data for α**-18**: R_*f*_ = 0.60 (ethyl acetate/toluene, 1/4, v/v); ^1^H NMR (400 MHz, CDCl_3_): δ 8.07 (d, 2H, *J* = 8.3 Hz, aromatic), 7.58–7.24 (m, 23H, aromatic), 5.59 (s, 1H, >C*H*Ph), 5.35 (dd, 1H, *J*_3’,4’_ = 3.1 Hz, H-3′), 5.13 (s, 1H, H-1′), 4.98 (d, 1H, ^2^ *J* = 11.2 Hz, C*H*Ph), 4.68 (d, 1H, ^2^ *J* = 12.0 Hz, C*H*Ph), 4.59–4.53 (m, 4H, H-1, 3 × C*H*Ph), 4.46 (d, 1H, ^2^ *J* = 11.2 Hz, C*H*Ph), 4.42–4.33 (m, 1H, H-5′), 4.15 (dd, 1H, *J*_6a’,6b’_ = 10.6 Hz, H-6a′), 4.06 (dd, 1H, *J*_4’,5’_ = 9.7 Hz, H-4′), 3.98 (d, 1H, *J*_2’, 3’_ = 3.2 Hz, H-2′), 3.75–3.68 (m, 5H, H-3, 5, 6a, 6b, 6 b’), 3.57–3.54 (m, 1H, H-4) 3.50 (dd, 1H, *J*_2,3_ = 9.2 Hz, H-2), and 3.31 (s, 3H, OCH_3_) ppm; ^13^C NMR (100 MHz, CDCl_3_): δ 165.7, 165.1, 139.3, 138.2 (×2), 138.0, 137.7, 137.5, 137.1, 137.0, 133.6, 133.3, 130.2, 129.9, 129.8 (×2), 129.7 (×2), 129.3, 129.2, 129.1 (×2), 128.8, 128.6 (×4), 128.5 (×2), 128.4 (×8), 128.2 (×5), 128.1 (×4), 127.9, 127.8, 127.7 (×2), 127.6 (×2), 127.5, 127.4 (×3), 127.2 (×2), 126.1 (×4), 102.2, 102.1, 100.2, 99.4, 98.2, 97.7, 81.4, 80.2 (×2), 79.5, 78.0, 77.9, 77.2, 75.4, 75.2, 74.1, 74.0, 73.8, 73.5, 73.2 (×2), 69.9, 69.7, 69.4, 69.0 (×2), 68.4, 68.1, 65.0, 61.4, 60.2, 59.9, 55.4, and 55.2 ppm; and HRMS [M + H]^+^ calcd for C_48_H_50_O_11_N_3_ 844.3443; found 844.3440.

Methyl 3-O-(2-azido-4,6-O-benzylidene-2-deoxy-3-O-picoloyl-D-altropyranosyl)-2,4,6-tri-O-benzyl-α-D-glucopyranoside **(19)**: the title compound was obtained as a pale yellow amorphous solid from glycosyl donor **9** and acceptor **13** in 63% yield (α/β = 2.2/1). Selected analytical data for α**-19**: R_*f*_ = 0.60 (ethyl acetate/toluene, 1/4, v/v); ^1^H NMR (400 MHz, CDCl_3_): δ 8.82 (dd, 1H, aromatic), 8.08 (dd, 1H, aromatic), 7.54–7.09 (m, 22H, aromatic), 5.58 (s, 1H, >C*H*Ph), 5.43 (dd, 1H, *J*_3’,4’_ = 3.1 Hz, H-3′), 5.11 (s, 1H, H-1′), 4.84–4.77 (m, 2H, H-5′, C*H*Ph), 4.55–4.46 (m, 3H, H-1, 2 x C*H*Ph,), 4.43–4.42 (m, 3H, 3 x C*H*Ph), 4.22 (dd, 1H, *J*_6’a,6’b_ = 10.4 Hz, H-6′a), 4.07 (dd, 1H, *J*_4,5_ = 9.9 Hz, H-4′), 4.00 (dd, 1H, *J*_3,4_ = 9.4 Hz, H-3), 3.93 (d, 1H, *J*_1’,2’_ = 3.0 Hz, H-2′), 3.76–3.63 (m, 3H, H-5, 6a, 6b′), 3.60 (d, 1H, H-6b), 3.50 (dd, 1H, *J*_4,5_ = 9.6 Hz, H-4), 3.31 (dd, 1H, *J*_2, 3_ = 9.7 Hz, H-2), and 3.28 (s, 3H, CH_3_) ppm; ^13^C NMR (100 MHz, CDCl_3_): δ 164.0, 150.4, 149.8, 147.9, 137.7, 137.6, 137.5 (×2), 137.4 (×4), 137.1, 136.9, 129.1, 129.0, 128.6 (×10), 128.5 (×2), 128.4 (×2), 128.2 (×8), 128.1 (×2), 128.0 (×3), 127.9 (×3), 127.7, 127.6, 127.4, 127.3 (×3), 127.2 (×3) 127.0 (×2), 126.3, 126.2, 126.1, 125.2 (×2), 102.2, 102.1, 99.1 (×2), 97.7, 97.5, 79.1, 78.9, 78.7, 78.2, 78.1, 77.6, 76.0, 74.6, 74.5, 73.9, 73.7 (×2), 73.2, 72.9, 69.8, 69.5, 69.4, 69.1, 69.0, 68.3, 68.1, 67.9, 61.9, 60.0, 59.2, 58.3, and 55.1 (×2) ppm; and HRMS [M + H]^+^ calcd for C_48_H_50_O_11_N_3_ 844.3443; found 844.3440.

Methyl 3-O-(2-azido-3-O-benzoyl-4,6-O-benzylidene-2-deoxy-D-altropyranosyl)-2,4,6-tri-O-benzyl-α-D-glucopyranoside **(20)**: the title compound was obtained as a pale yellow amorphous solid from glycosyl donor **10** and acceptor **13** in 80% yield (α/β = 2.5/1). Selected analytical data for α**-20**: R_*f*_ = 0.60 (ethyl acetate/toluene, 1/4, v/v); ^1^H NMR (400 MHz, CDCl_3_): δ 8.17–8.10 (m, 2H, aromatic), 7.64–7.60 (m, 1H, aromatic), 7.48–7.11 (m, 22H, aromatic), 5.57 (s, 1H, >C*H*Ph), 5.37 (dd, 1H, *J*_3’,4’_ = 3.0 Hz, H-3′), 5.09 (s, 1H, H-1′), 4.76 (m, 2H, H-5′, C*H*Ph), 4.60–4.41 (m, 6H, H-1, 5 x C*H*Ph,), 4.19 (dd, 1H, *J*_6’_a,6b = 10.3 Hz, H-6′a), 4.05 (dd, 1H, *J* = 10.0 Hz, H-4′), 3.99 (dd, 1H, *J*_3,4_ = 9.4 Hz, H-3), 3.91 (d, 1H, *J*_2’,3’_ = 2.9 Hz, H-2′), 3.76–3.54 (m, 4H, H-5, 6a, 6b, 6b′), 3.48 (dd, 1H, *J*_4,5_ = 9.5 Hz, H-4), 3.32 (d, 1H, *J*_2,3_ = 9.7 Hz, H-2), and 3.26 (s, 3H, CH_3_) ppm; ^13^C NMR (100 MHz, CDCl_3_): δ 165.9, 137.7, 137.5 (×2), 137.4, 133.4, 130.2, 129.8 (×3), 129.0, 128.6 (×4), 128.5 (×4), 128.4 (×3), 128.2 (×2), 128.1 (×3), 128.0, 127.9, 127.4, 126.3, 102.1, 99.3, 97.8, 79.4, 78.7, 78.4, 77.2, 74.6, 74.0, 73.7, 73.3, 69.6, 69.1, 68.7, 68.2, 60.2, 59.3, and 55.1 ppm; and HRMS [M + H]^+^ calcd for C_48_H_50_O_11_N_3_ 844.3443; found 844.3440.

Methyl 2-O-(2-azido-4,6-O-benzylidene-2-deoxy-3-O-picoloyl-α-D-altropyranosyl)-3,4,6-tri-O-benzyl-α-D-glucopyranoside **(21)**: the title compound was obtained as a pale yellow amorphous solid from glycosyl donor **9** and acceptor **14** ^29^ in 65% yield. Analytical data for **21**: R_*f*_ = 0.40 (ethyl acetate/toluene, 3/7, v/v); [α]_D_^24^ +69.9 (*c* 1.0, CHCl_3_); ^1^H NMR (400 MHz, CDCl3): δ 8.56 (dd, 1H, aromatic), 7.92 (d, 1H, aromatic), 7.46–6.97 (m, 22H, aromatic), 5.63 (s, 1H, >CHPh), 5.49 (dd, 1H, *J*_3’,4’_ = 3.1 Hz, H-3′), 4.95 (d, 1H, C*H*Ph) 4.93 (s, 1H, H-1′), 4.83 (d, 1H, *J*_1,2_ = 3.4 Hz, H-1), 4.75–4.65 (m, 2H, *J*_5’,6’a_ = 5.4 Hz, H-5′, C*H*Ph), 4.59 (d, 1H, C*H*Ph), 4.52–4.38 (m, 3H, 3 × C*H*Ph), 4.29 (dd, 1H, *J*_6’a,6’b_ = 10.4 Hz, H-6′a), 4.26 (d, 1H, *J*_2’,3’_ = 2.8 Hz, H-2′), 4.11 (dd, 1H, *J*_4’,5’_ = 9.9 Hz, H-4′), 3.88 (dd, 1H, *J*_2,3_ = 9.6 Hz, H-2), 3.78 (dd, 1H, *J*_3,4_ = 9.7 Hz, H-3), 3.76 (dd, 1H, H-6′b) 3.71–3.60 (m, 4H, H-4, 5, 6a, 6b), and 3.29 (s, 3H, CH_3_) ppm; ^13^C NMR (100 MHz, CDCl_3_): δ 164.3, 149.5, 147.1, 137.9, 137.6, 137.4, 136.8, 136.2, 128.7, 128.0, 127.9 (×4), 127.8 (×5), 127.6 (x2), 127.4 (×2), 127.3 (×3), 127.2, 127.1, 126.4, 125.8, 124.9, 101.8, 96.0, 95.6, 80.0, 77.3, 76.3, 75.0, 74.5, 73.3, 73.1, 69.5, 68.9, 68.6, 67.9, 60.1, 58.9, and 54.5 ppm; and HRMS [M + Na]^+^ calcd for C_47_H_48_N_4_O_11_Na 867.3238; found 867.3212.

Methyl 2-O-(2-azido-3-O-benzoyl-4,6-O-benzylidene-2-deoxy-D-altropyranosyl)-3,4,6-tri-O-benzyl-α-D-glucopyranoside **(22)**: the title compound was obtained as a yellowish sticky semi-solid from glycosyl donor **10** and acceptor **14** in 66% yield (α/β = 1.5/1). Selected analytical data for α**-22**: R_*f*_ = 0.60 (ethyl acetate/toluene, 1/4, v/v); ^1^H NMR (400 MHz, CDCl_3_): δ 8.01–7.93 (m, 2H, aromatic), 7.52–6.98 (m, 23H, aromatic), 5.61 (s, 1H, >C*H*Ph), 5.39 (dd, 1H, *J*_3’,4’_ = 3.1 Hz, H-3′), 4.94 (s, 1H, H-1′), 4.87–4.84 (d, 1H, C*H*Ph), 4.79 (d, 1H, *J*_1,2_ = 3.3 Hz, H-1), 4.68 (dd, 1H, *J*_5’,6’a_ = 4.7, *J*_5’,6’b_ = 10.4 Hz, H-5′), 4.60–4.28 (m, 5H, 5 × C*H*Ph), 4.29 (dd, 1H, *J*_6’a,6’b_ = 10.5 Hz, H-6′a), 4.25 (d, 1H, *J*_2’,3’_ = 2.7 Hz, H-2′), 4.09 (dd, 1H, *J*_4’,5’_ = 9.8 Hz, H-4′), 3.84 (dd, 1H, *J*_2,3_ = 9.6 Hz, H-2), 3.81–3.75 (m, 2H, H-3, 6′b), 3.72–3.58 (m, 4H, H-4, 5, 6a, 6 b), and 3.24 (s, 3H, CH_3_) ppm; ^13^C NMR (100 MHz, CDCl_3_): δ 166.5, 165.2, 138.3, 138.1, 137.9, 137.8, 137.2, 136.9, 133.7, 133.5, 133.2, 129.9, 129.8 (×4), 129.7, 129.6, 129.2 (×3), 128.7, 128.6, 128.5, 128.4 (×8), 128.3 (×6), 128.2 (×2), 128.0, 127.9 (×8), 127.8 (×3), 127.7 (x2), 127.6 (x2), 127.5, 126.3 (x2), 126.1 (x2), 126.0, 102.4, 102.2, 96.7, 96.6, 93.0, 92.5, 80.5, 77.8, 77.1, 75.5, 75.1, 75.0, 74.1, 74.0, 73.8, 73.5 (×2), 70.0, 69.5, 69.2, 69.1, 68.9 (×2), 68.4, 68.3, 65.0, 62.3, 60.5, 60.4, 60.3, 59.5, 59.3, and 54.9 ppm; and HRMS [M + H]^+^ calcd for C_48_H_50_O_11_N_3_ 844.3443; found 844.3440.

## Preparation of disaccharide 23 to understand stereoselectivity

Methyl 6-O-(2-azido-4,6-O-benzylidene-2-deoxy-α-D-altropyranosyl)-2,3,4-tri-O-benzyl-α-D-glucopyranoside **(23)** from compound **15**: Copper acetate monohydrate (12.4 mg, 0.070 mmol) was added to a solution of compound **15** (38 mg, 0.05 mmol) in CH_2_Cl_2_/MeOH (1.2 ml, 3/1, v/v), and the resulting mixture was stirred for 1 h at room temperature. After that, the reaction mixture was diluted with CH_2_Cl_2_ (∼30 ml) and washed with water (10 ml), sat. aq. NaHCO_3_ (10 ml), and water (2 × 10 ml). The organic phase was separated, dried with Na_2_SO_4_, and concentrated under reduced pressure. The residue was purified by column chromatography on silica gel (ethyl acetate–hexane gradient elution) to afford the title compound as a white amorphous solid. Analytical data for **23**: R_*f*_ = 0.40 (ethyl acetate/toluene, 3/7, v/v); [α]_D_^22^ +8.8 (*c* 1.0, CHCl_3_); ^1^H NMR (400 MHz, CDCl_3_): δ 7.46 (dd, 2H, aromatic), 7.41–7.21 (m, 18H, aromatic), 5.61 (s, 1H, >C*H*Ph), 5.01–4.93 (m, 2H, 2 × C*H*Ph), 4.86 (s, 1H, H-1′), 4.82–4.60 (m, 4H, 4 × C*H*Ph), 4.61 (d, 1H, *J*_1,2_ = 2.1 Hz, H-1), 4.25 (dd, 1H, *J*_5’,6’a_ = 5.1, *J*_5,6’b_ = 10.0 Hz, H-5′), 4.19–4.11 (m, 2H, H-3′, 6′a), 4.01 (dd, 1H, *J*_3,4_ = 9.2 Hz, H-3), 3.93 (d, 1H, *J*_2’,3’_ = 3.1 Hz, H-2′), 3.85 (dd, 1H, *J*_4’,5’_ = 9.7 Hz, H-4′), 3.83–3.76 (m, 3H, H-5, 6a, 6 b′), 3.64 (dd, 1H, H-6b), 3.52 (dd, 1H, *J*_2,3_ = 9.6 Hz, H-2), 3.42 (br d, 1H, *J*_4,5_ = 9.0 Hz, H-4), 3.37 (s, 3H, CH_3_), and 3.02 (br d, 1H, *J* = 7.9 Hz, OH) ppm; ^13^C NMR (100 MHz, CDCl_3_): δ 138.1, 137.6 (×2), 136.6, 128.7, 128.1 (×2), 128.0 (×2), 127.8 (×4), 127.7 (×2), 127.6, 127.5 (×2), 127.4, 127.3 (×2), 127.2, 125.8 (×2), 101.9, 98.0, 97.5, 81.5, 79.5, 77.5, 75.5, 75.3, 74.6, 72.9, 69.0, 68.5, 67.3, 66.7, 61.3, 58.0, and 54.9 ppm; and HRMS [M + Na]^+^ calcd for C_41_H_45_N_3_O_10_Na 867.3238; found 867.3212.

From compound **16**, a 1 N solution of sodium methoxide in MeOH was added to a solution of compound **15** (38 mg, 0.05 mmol) in MeOH (1.2 ml) until pH reached ∼ 9, and the resulting mixture was kept for 1 h at room temperature. After that, the reaction mixture was neutralized with Dowex (H^+^); the resin was filtered off and washed successively with MeOH. The combined filtrate (∼25 ml) was concentrated under reduced pressure. The residue was purified by column chromatography on silica gel (ethyl acetate–hexane gradient elution) to afford the title compound as a white amorphous solid.

## Data Availability

The datasets presented in this study can be found in online repositories. The names of the repository/repositories and accession number(s) can be found in the article/Supplementary Material.
