# Supplementary material for: Synthesis and glycosidation of building blocks of D-altrosamine
Source: Front Chem. 2022 Sep 26;10:945779. doi: 10.3389/fchem.2022.945779 (PMC9548543; doi:10.3389/fchem.2022.945779)
Supplement: Supplementary file 1 [file DataSheet1.PDF]

Supporting Information

Synthesis and glycosidation of building blocks of D-altrosamine

Mariya Novakova,[a] Anupama Das,[a] Catherine Alex,[b] and Alexei V. Demchenko[a,b]*

[a] Department of Chemistry, Saint Louis University, 3501 Laclede Ave, St. Louis, Missouri 63103, USA; e-mail: alexei.demchenko@slu.edu

[b] Department of Chemistry and Biochemistry, University of Missouri – St. Louis, One University Boulevard, St. Louis, Missouri 63121, USA;

Contents:

NMR Spectra for New Compounds ………………………………………………… S2

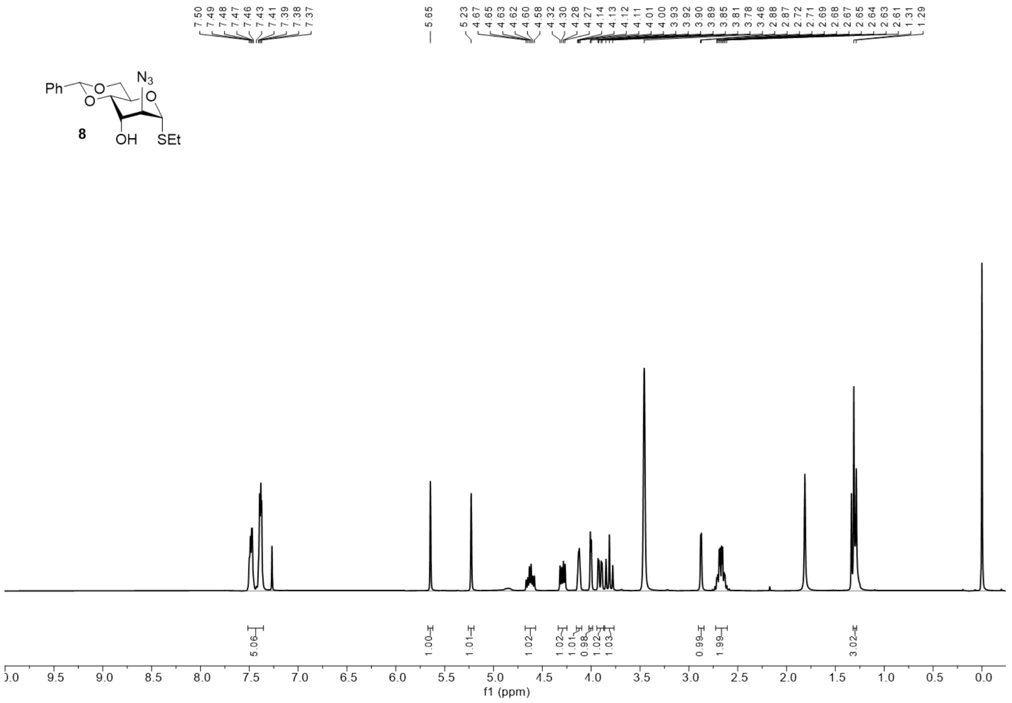

Figure S1. ^{1}H NMR spectrum of **8** ($CDCl_3$, 400 MHz).

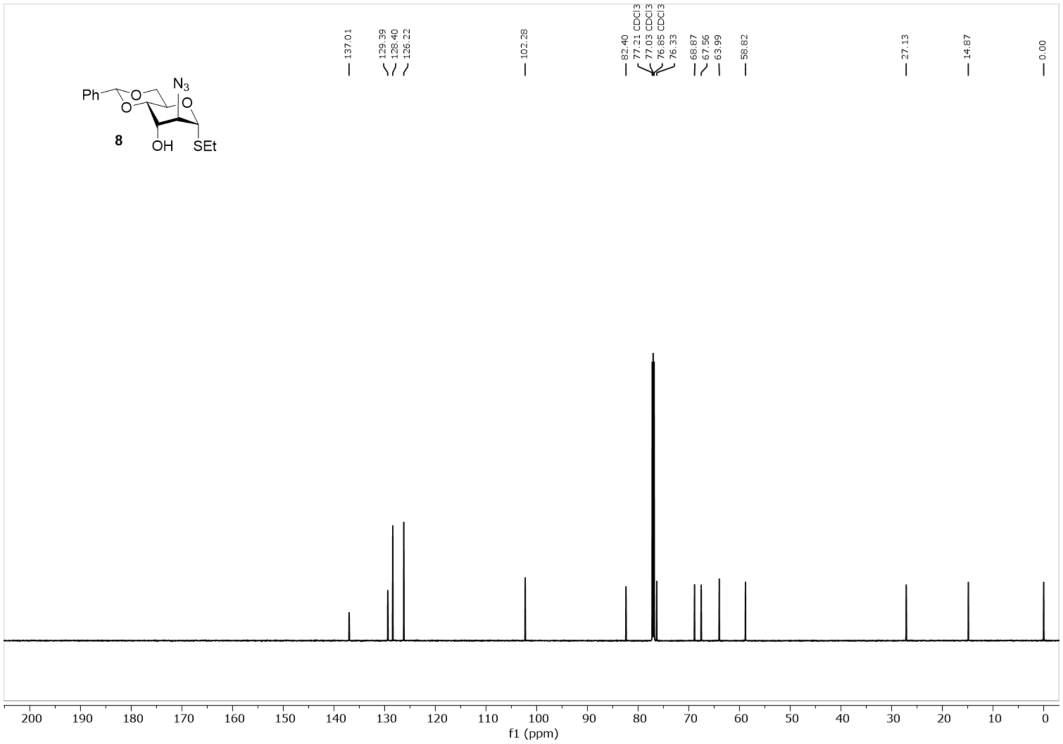

Figure S2. ^{13}C NMR spectrum of **8** ($CDCl_3$, 100 MHz).

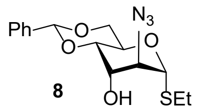

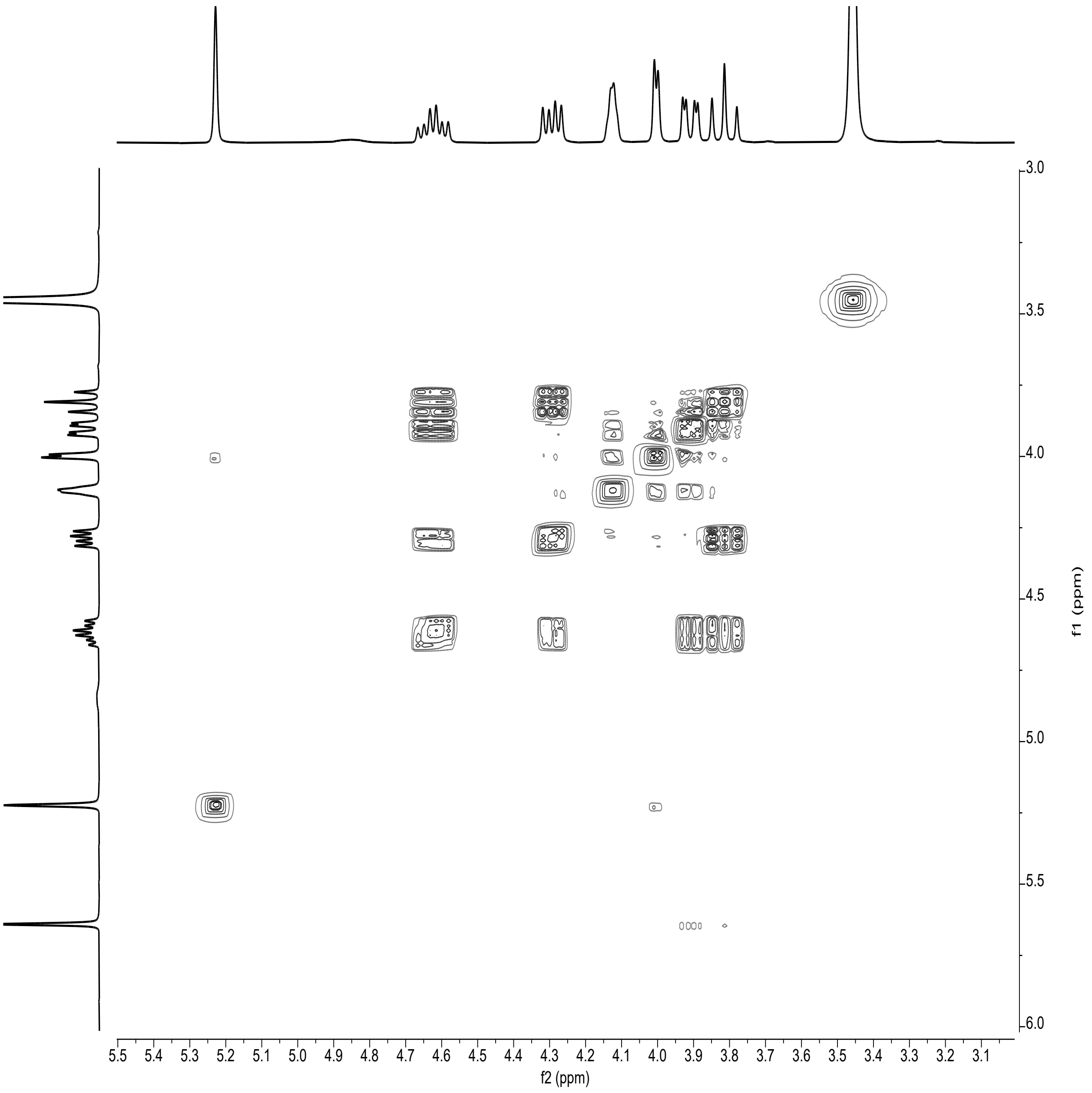

Figure S3. ^{1}H-^{1}H COSY spectrum of **8** ($CDCl_3$, 400 MHz).

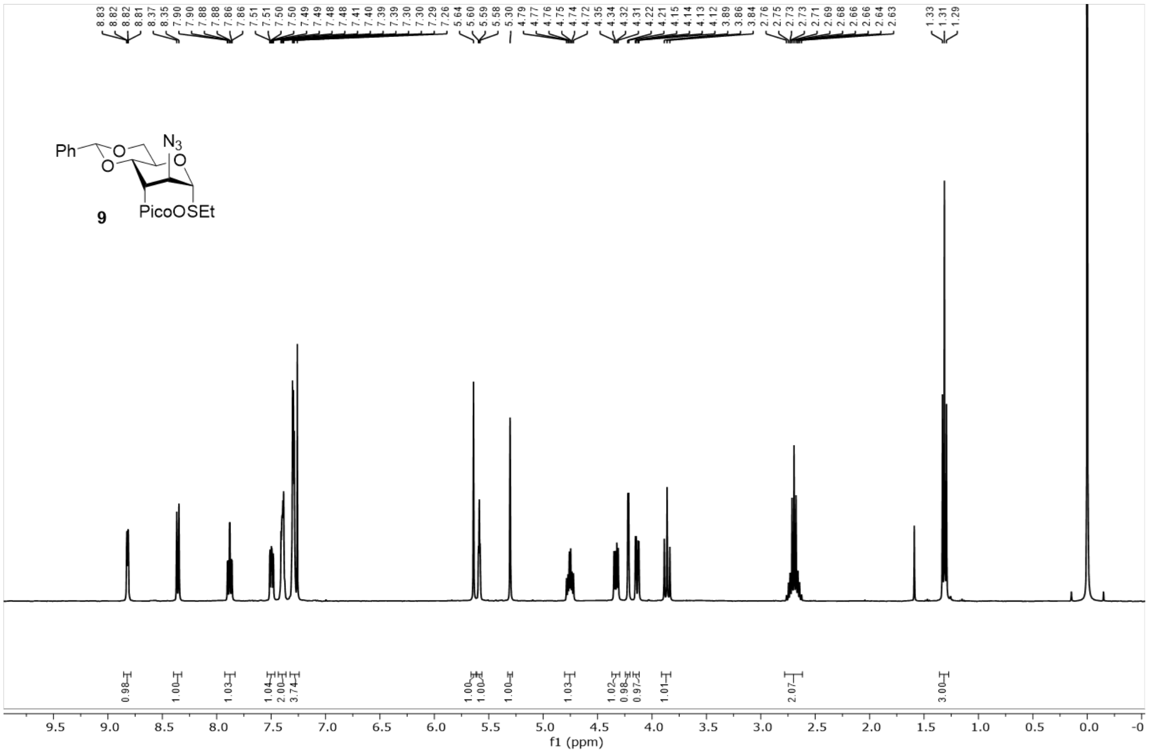

Figure S4. ^{1}H NMR spectrum of **9** ($CDCl_3$, 400 MHz).

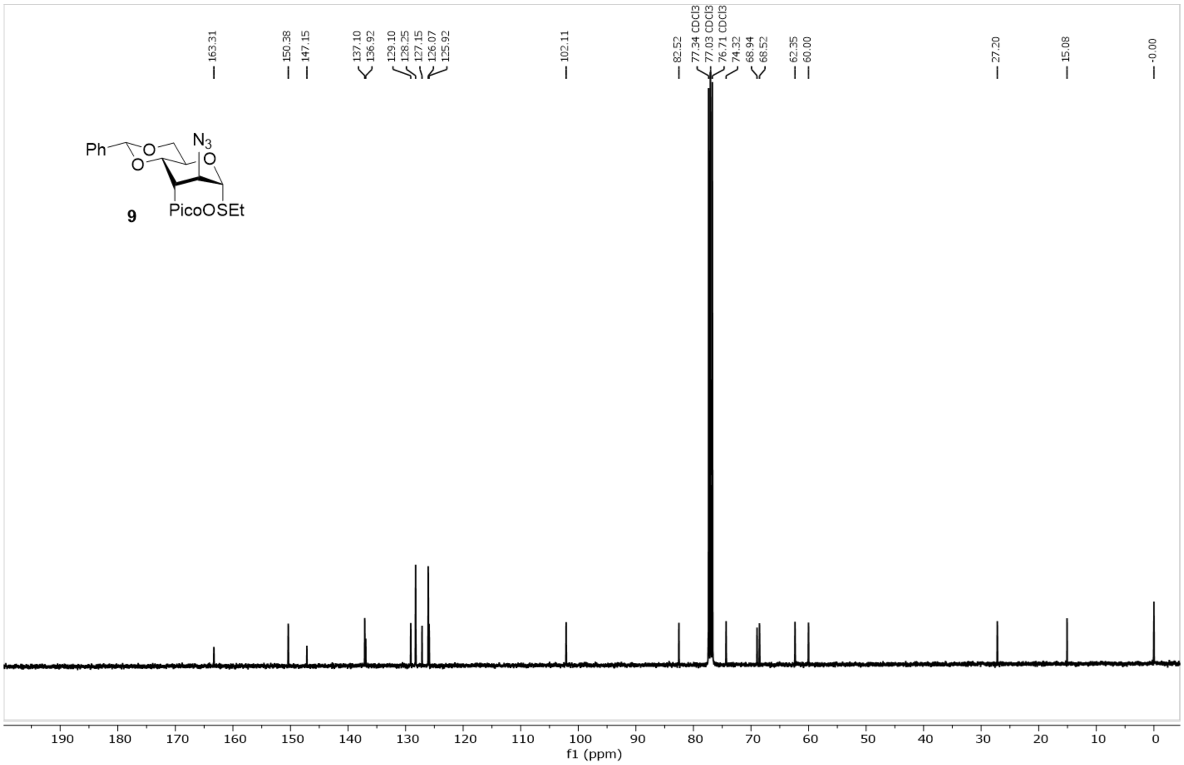

Figure S5. ^{13}C NMR spectrum of **9** ($CDCl_3$, 100 MHz).

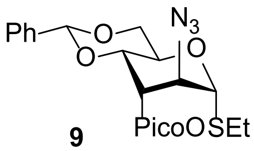

9

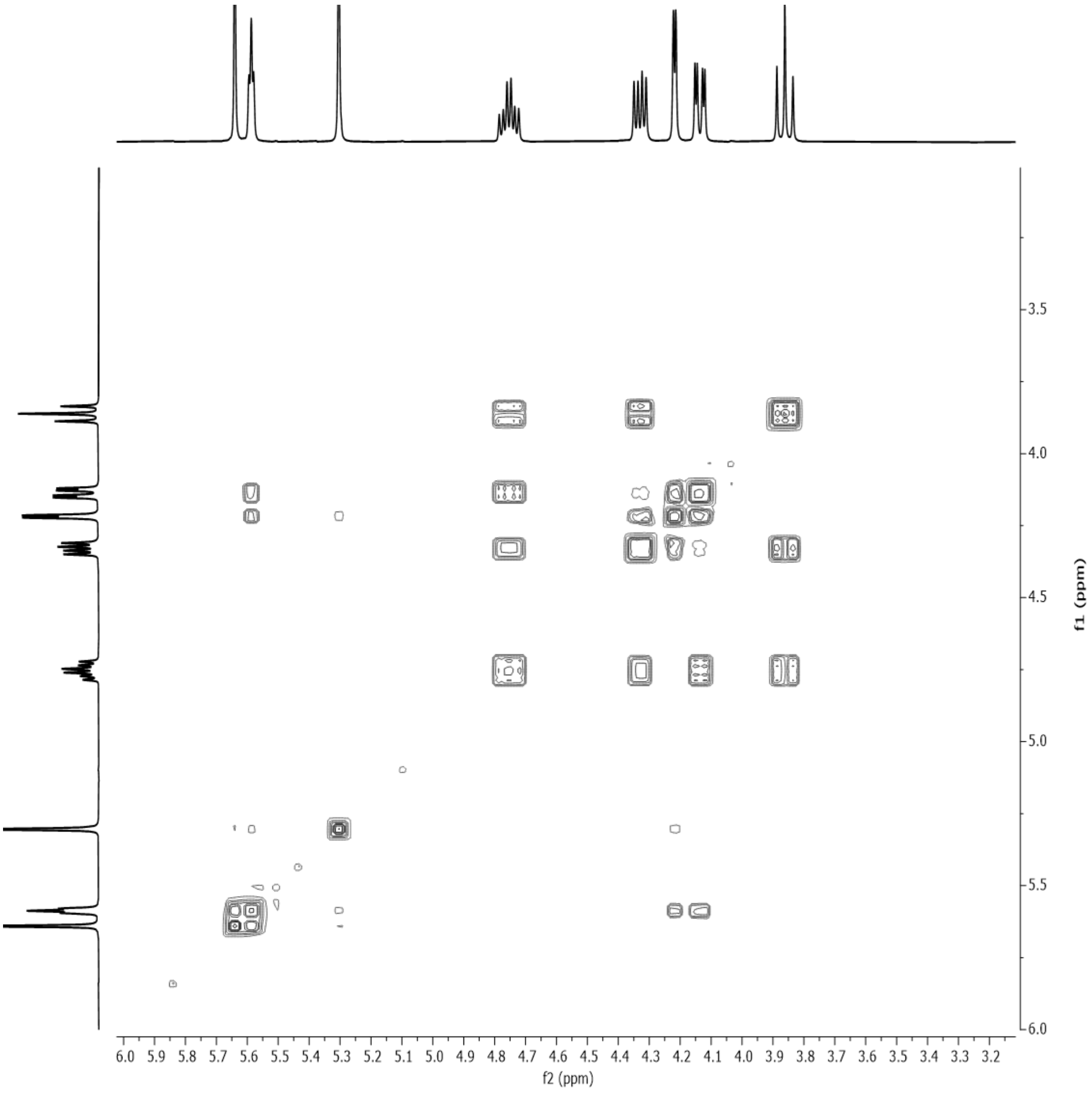

Figure S6. ^{1}H-^{1}H COSY spectrum of **9** ($CDCl_3$, 400 MHz).

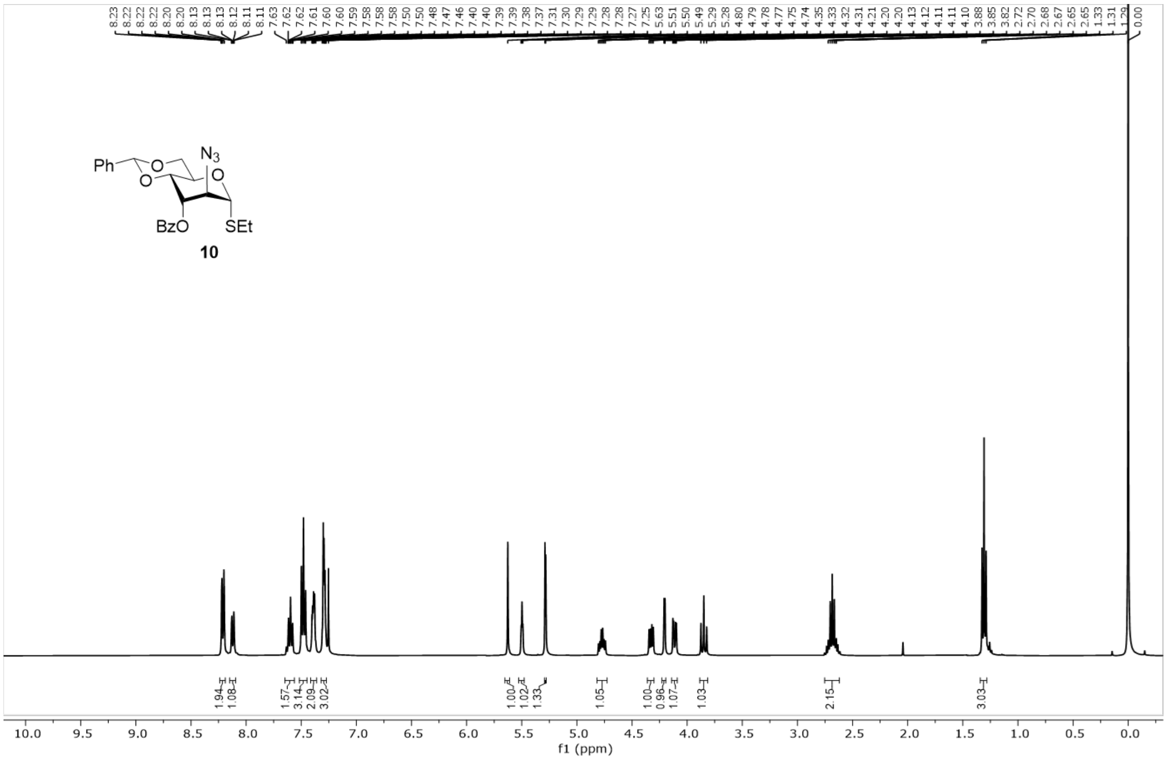

Figure S7. ^{1}H NMR spectrum of **10** ($CDCl_3$, 400 MHz).

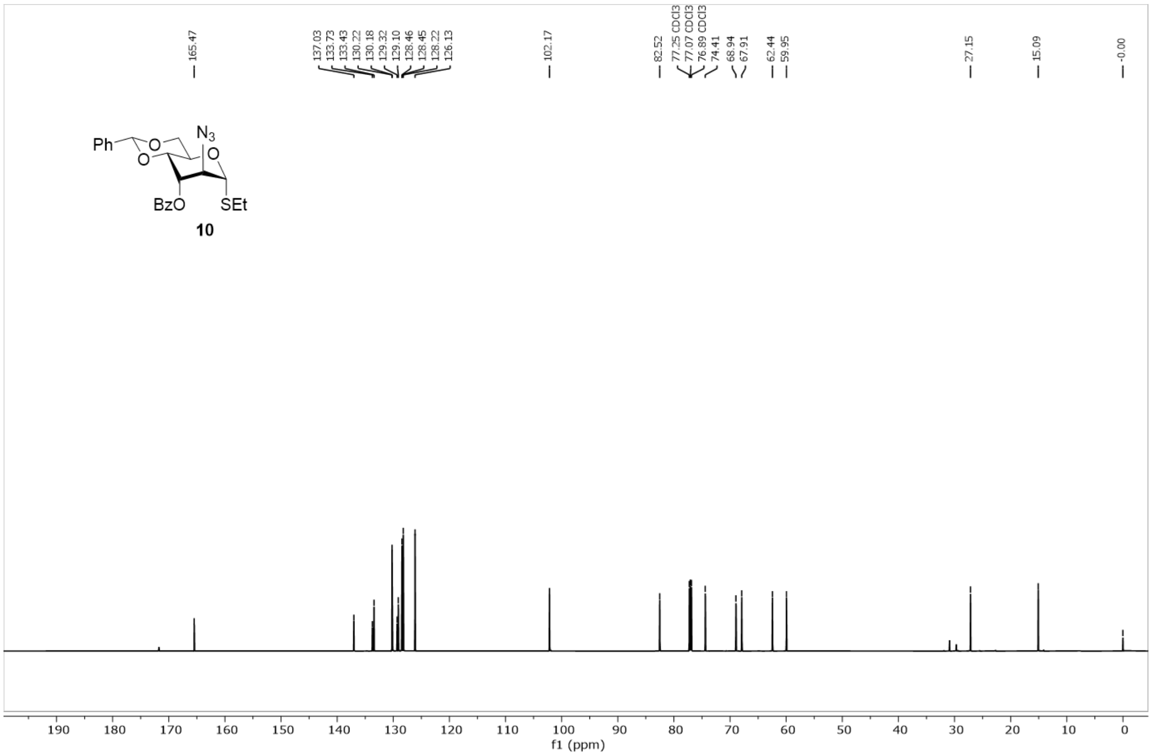

Figure S8. ^{13}C NMR spectrum of **10** ($CDCl_3$, 100 MHz).

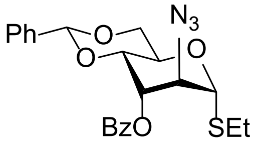

10

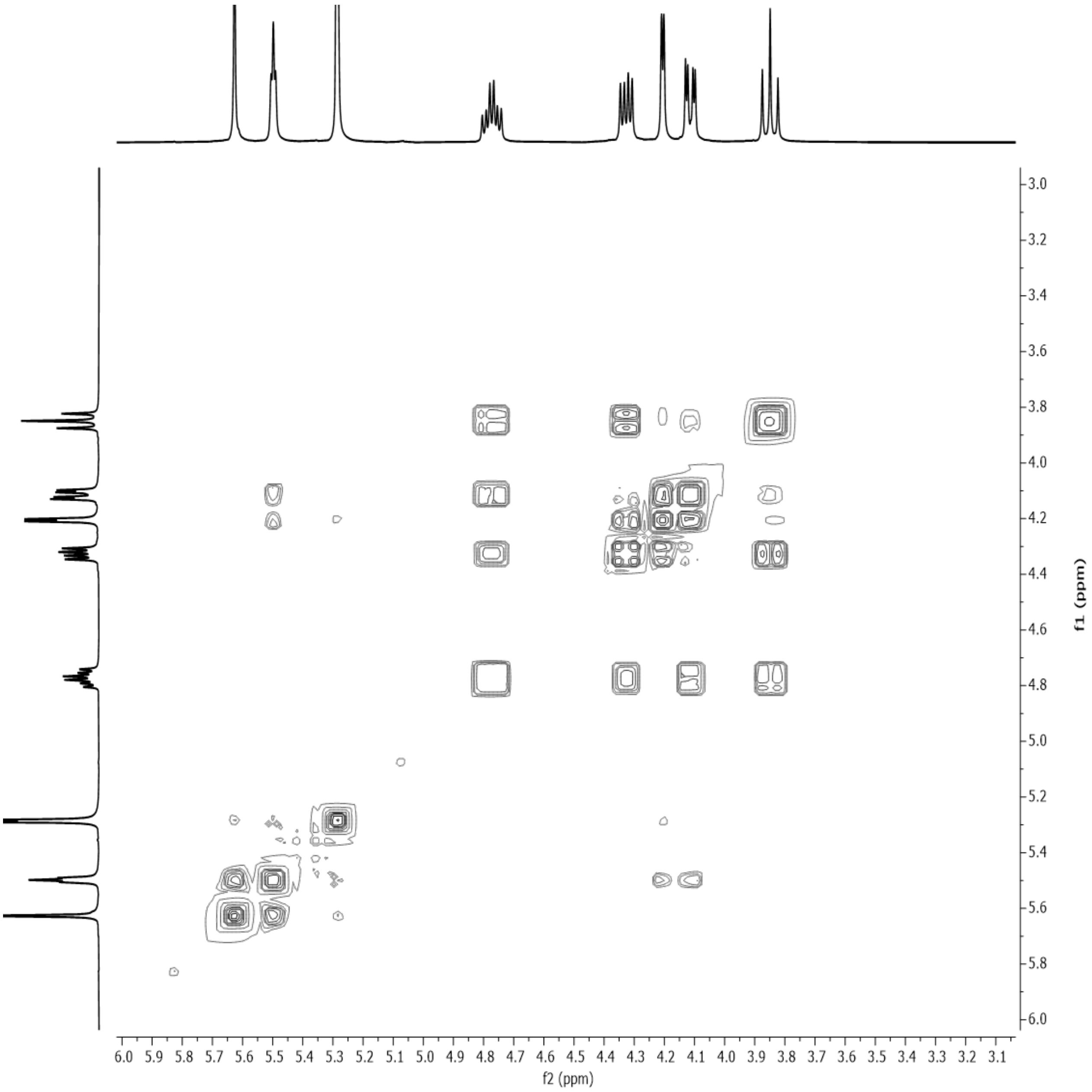

Figure S9. ^{1}H-^{1}H COSY spectrum of **10** ($CDCl_3$, 400 MHz).

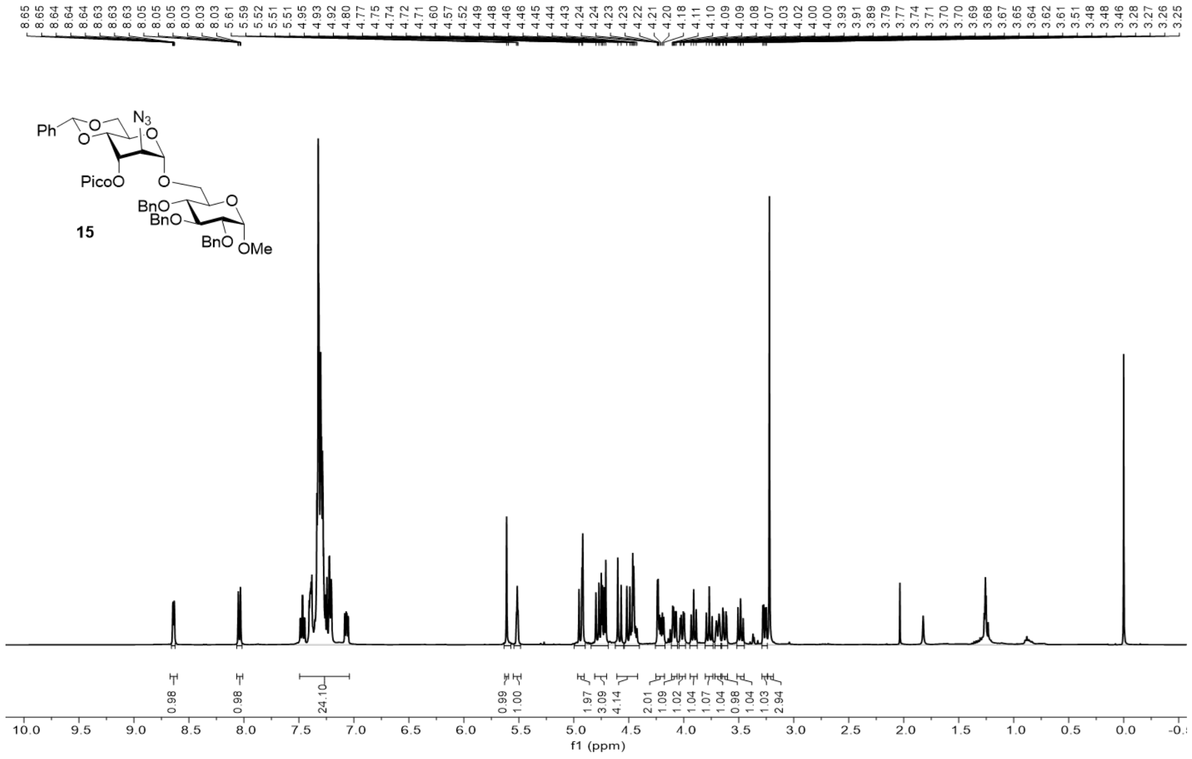

Figure S10. ^{1}H NMR spectrum of **15** ($CDCl_3$, 400 MHz).

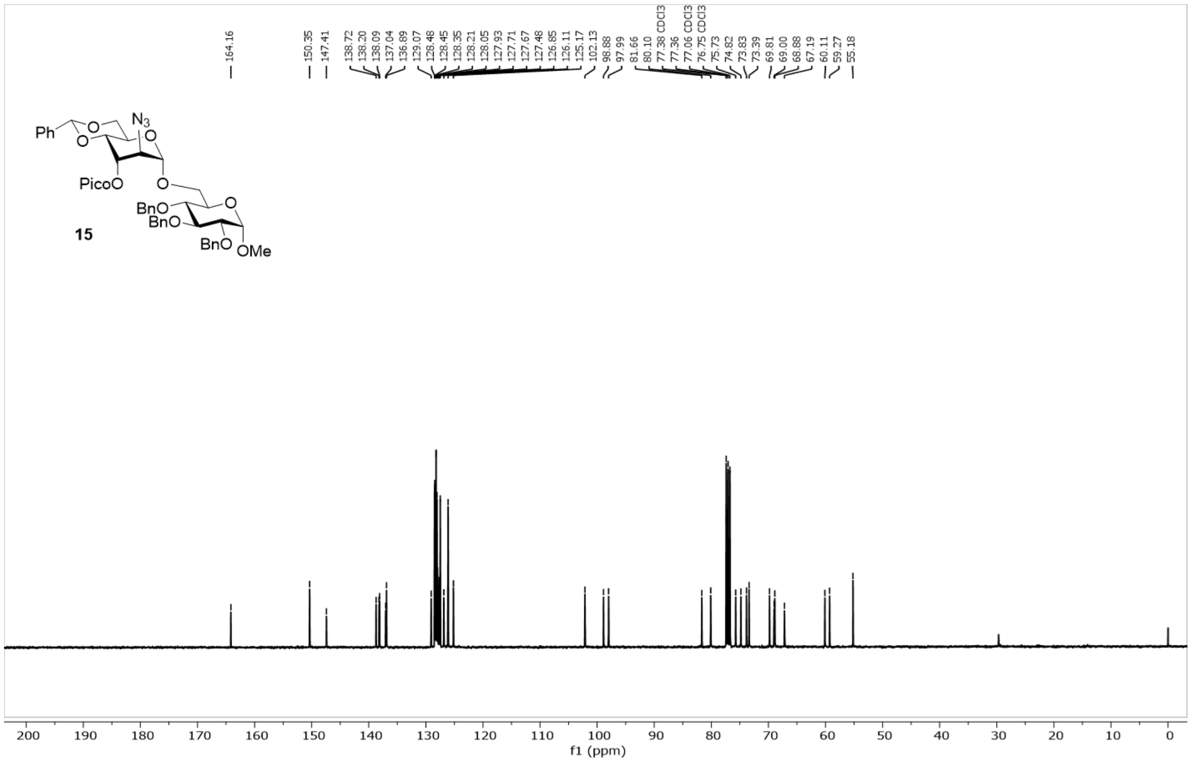

Figure S11. ^{13}C NMR spectrum of **15** ($CDCl_3$, 100 MHz).

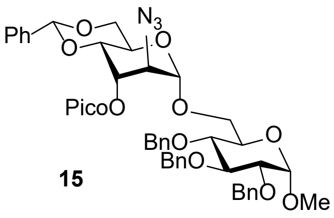

15

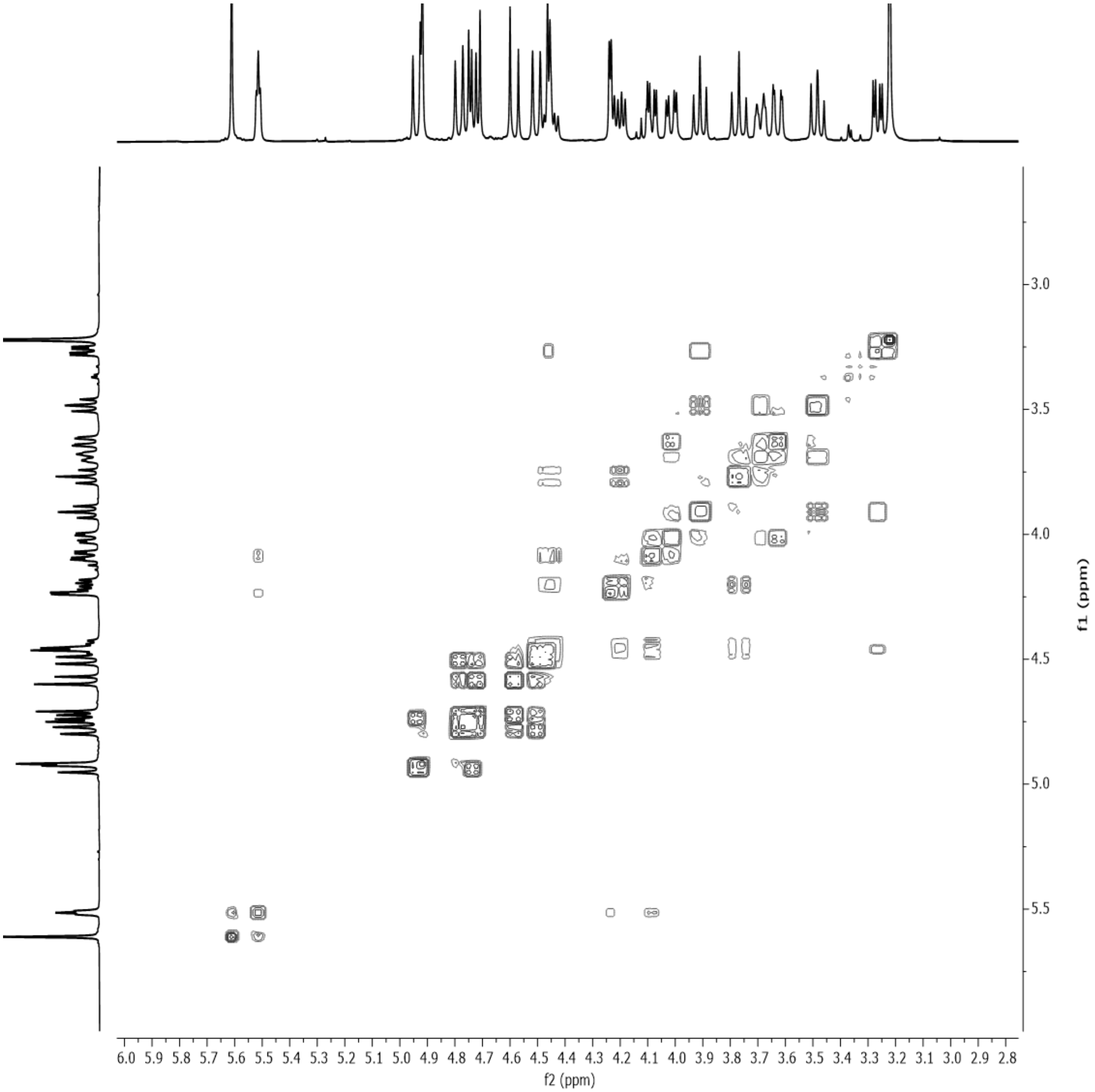

Figure S12. ^{1}H-^{1}H COSY spectrum of **15** ($CDCl_3$, 400 MHz).

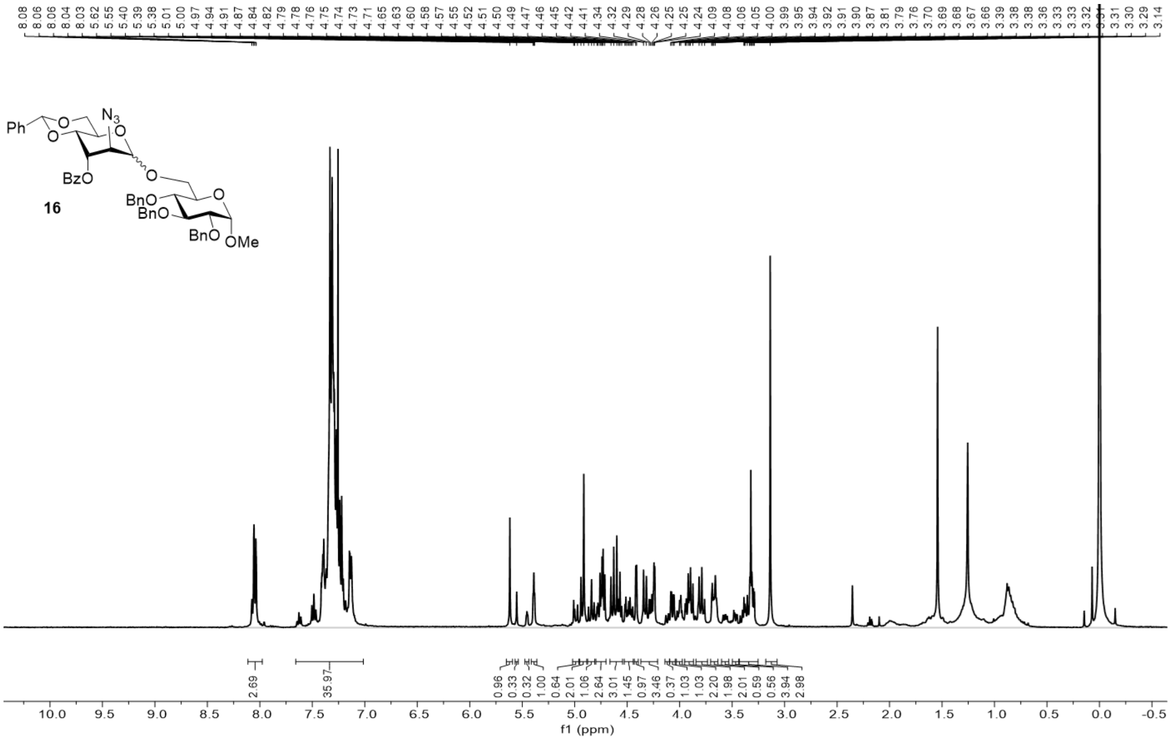

Figure S13. ^{1}H NMR spectrum of **16** ($CDCl_3$, 400 MHz).

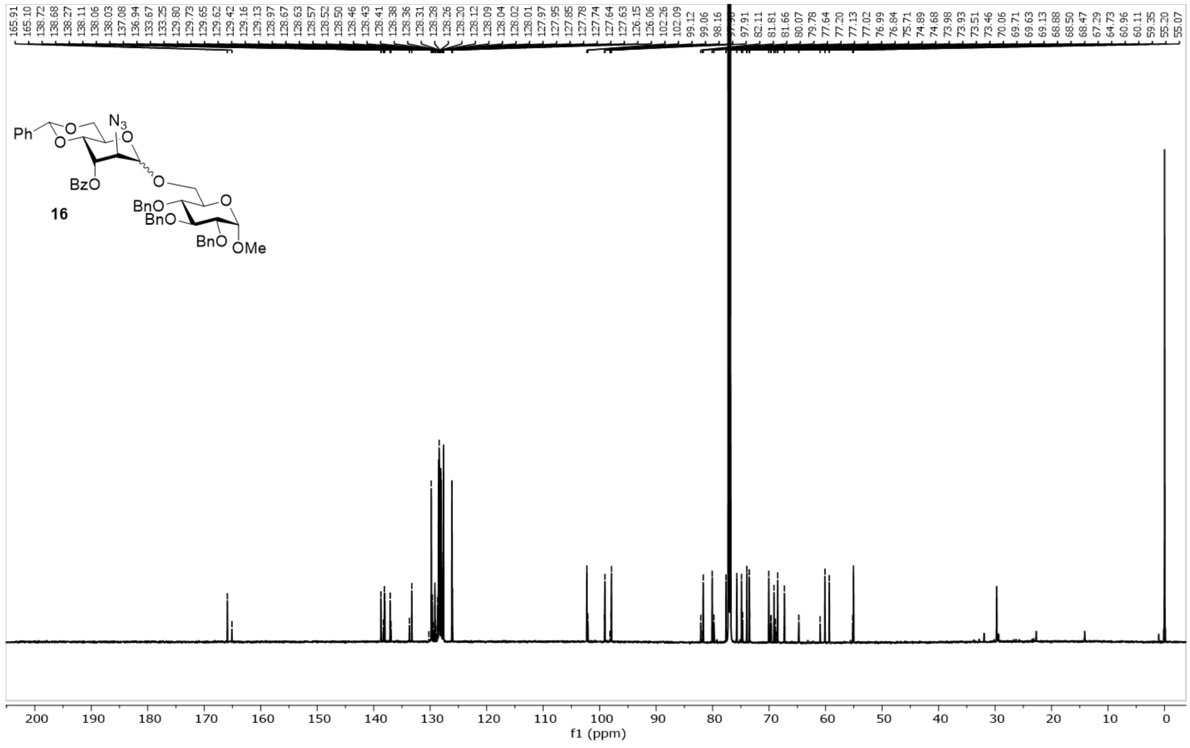

Figure S14. ^{13}C NMR spectrum of **16** ($CDCl_3$, 100 MHz).

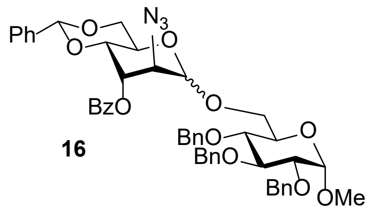

16

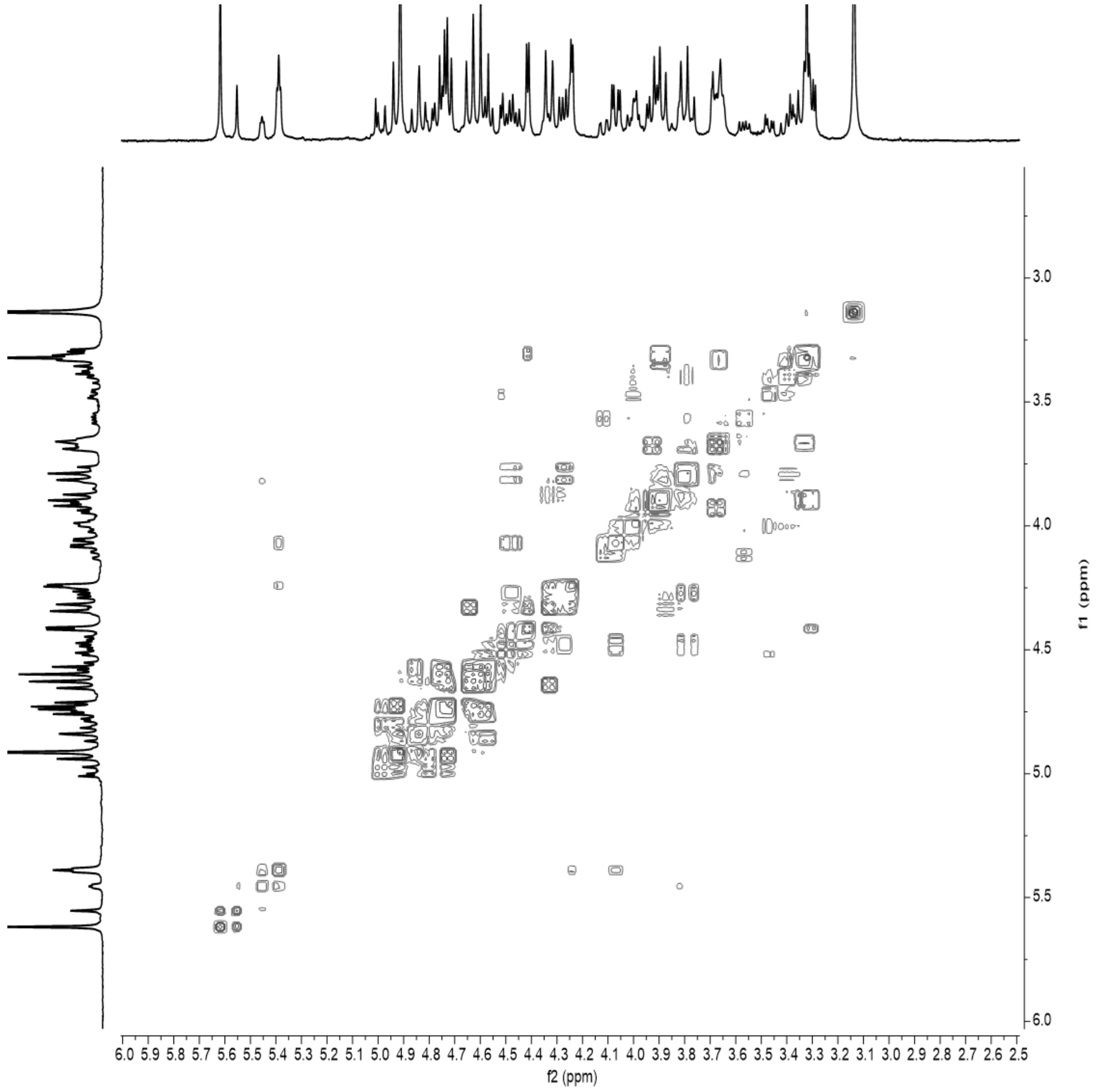

Figure S15. ^{1}H-^{1}H COSY spectrum of **16** ($CDCl_3$, 400 MHz).

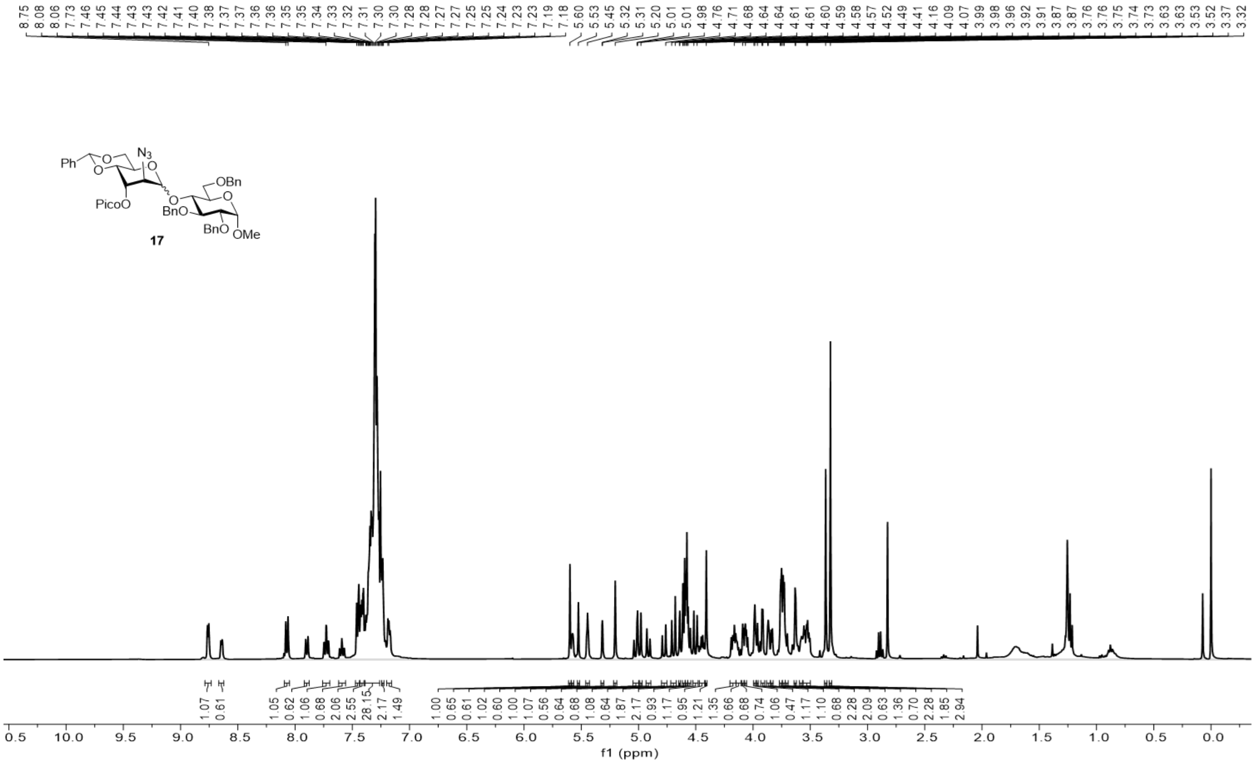

Figure S16. ^{1}H NMR spectrum of **17** ($CDCl_3$, 400 MHz).

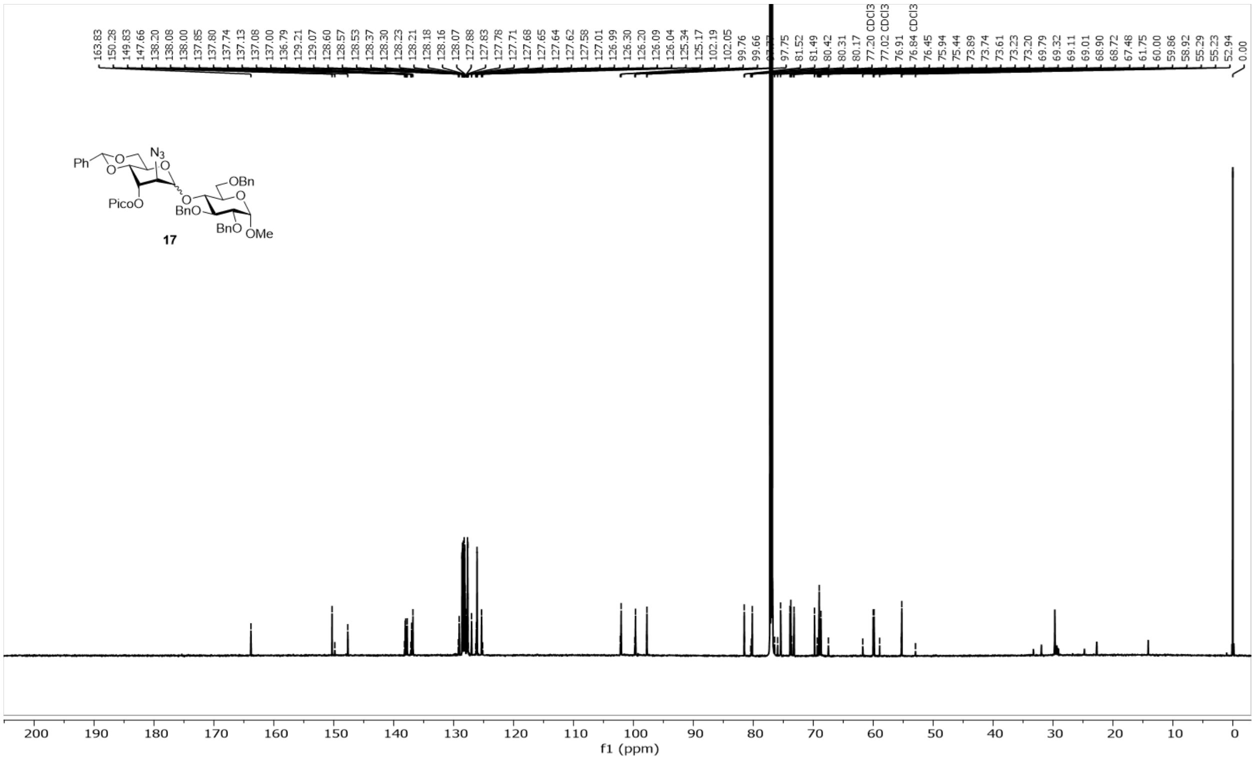

Figure S17. ^{13}C NMR spectrum of **17** ($CDCl_3$, 100 MHz).

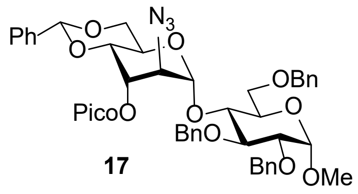

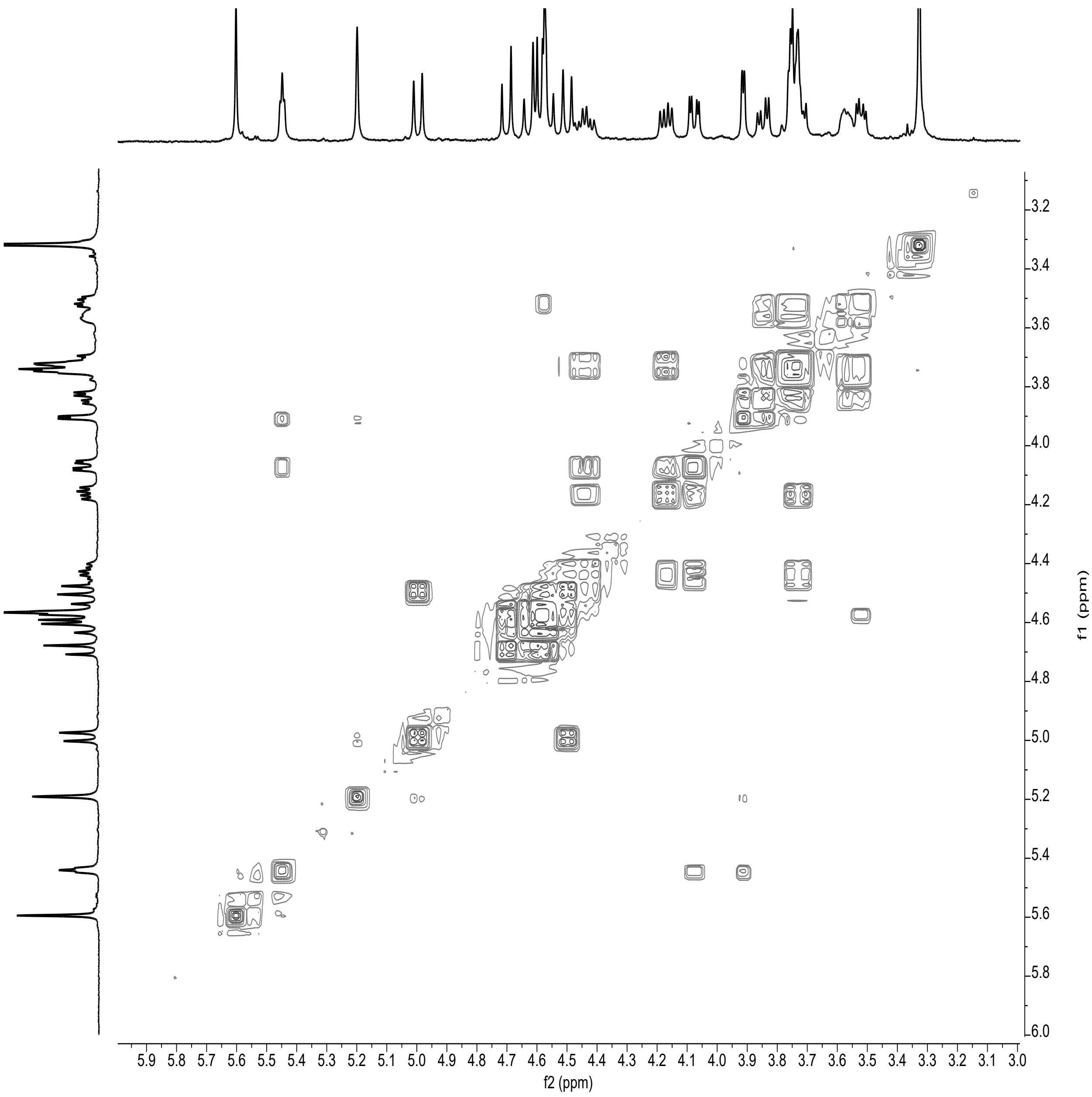

Figure S18. ^{1}H-^{1}H COSY spectrum of **17** ($CDCl_3$, 400 MHz).

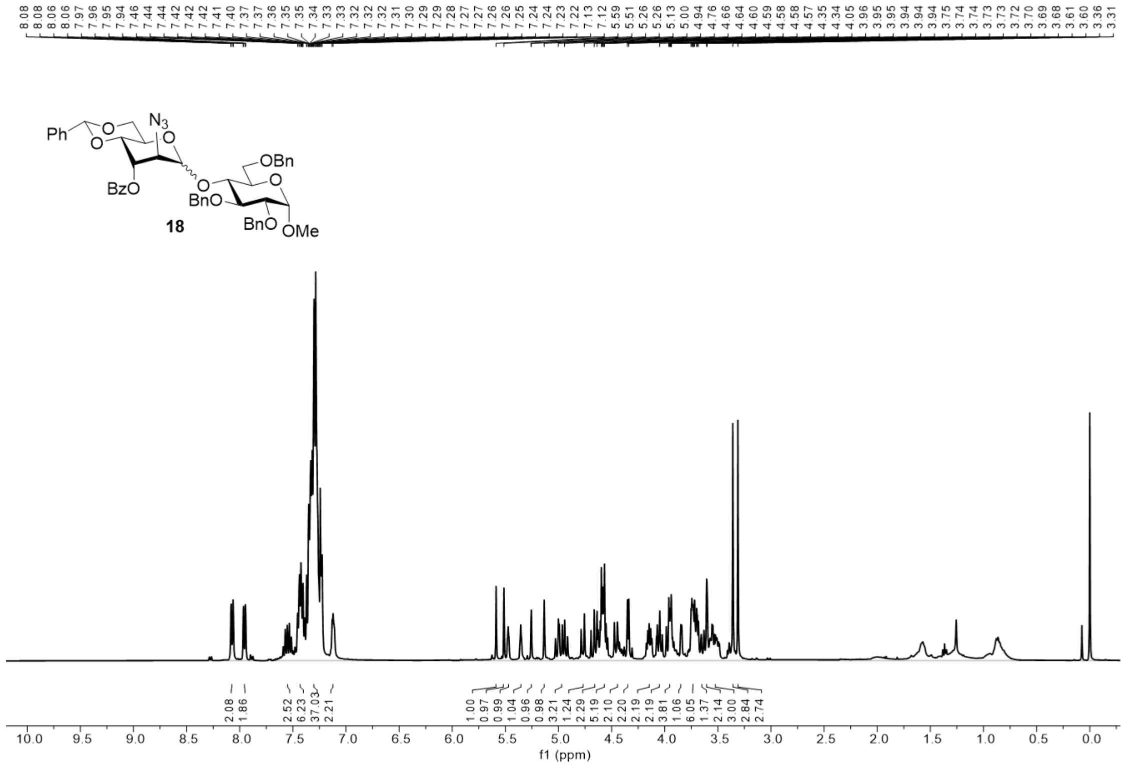

Figure S19. ^{1}H NMR spectrum of **18** ($CDCl_3$, 400 MHz).

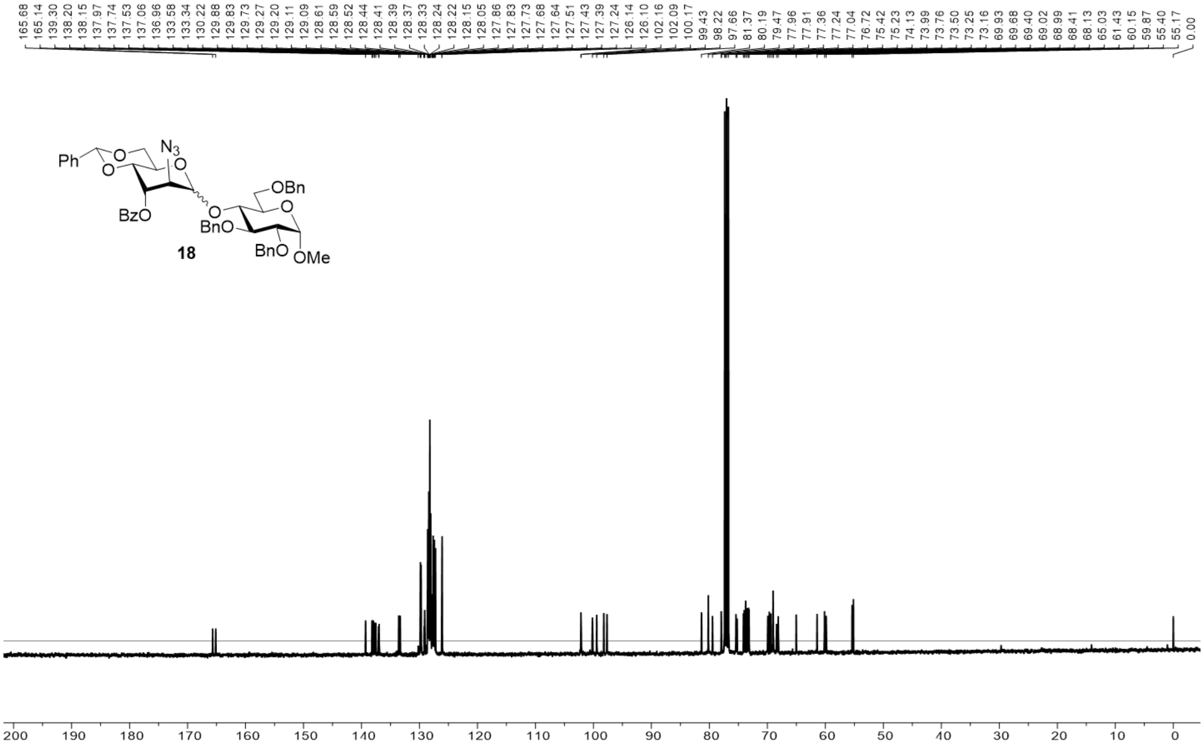

Figure S20. ^{13}C NMR spectrum of **18** ($CDCl_3$, 100 MHz).

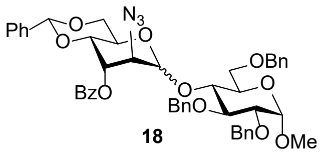

18

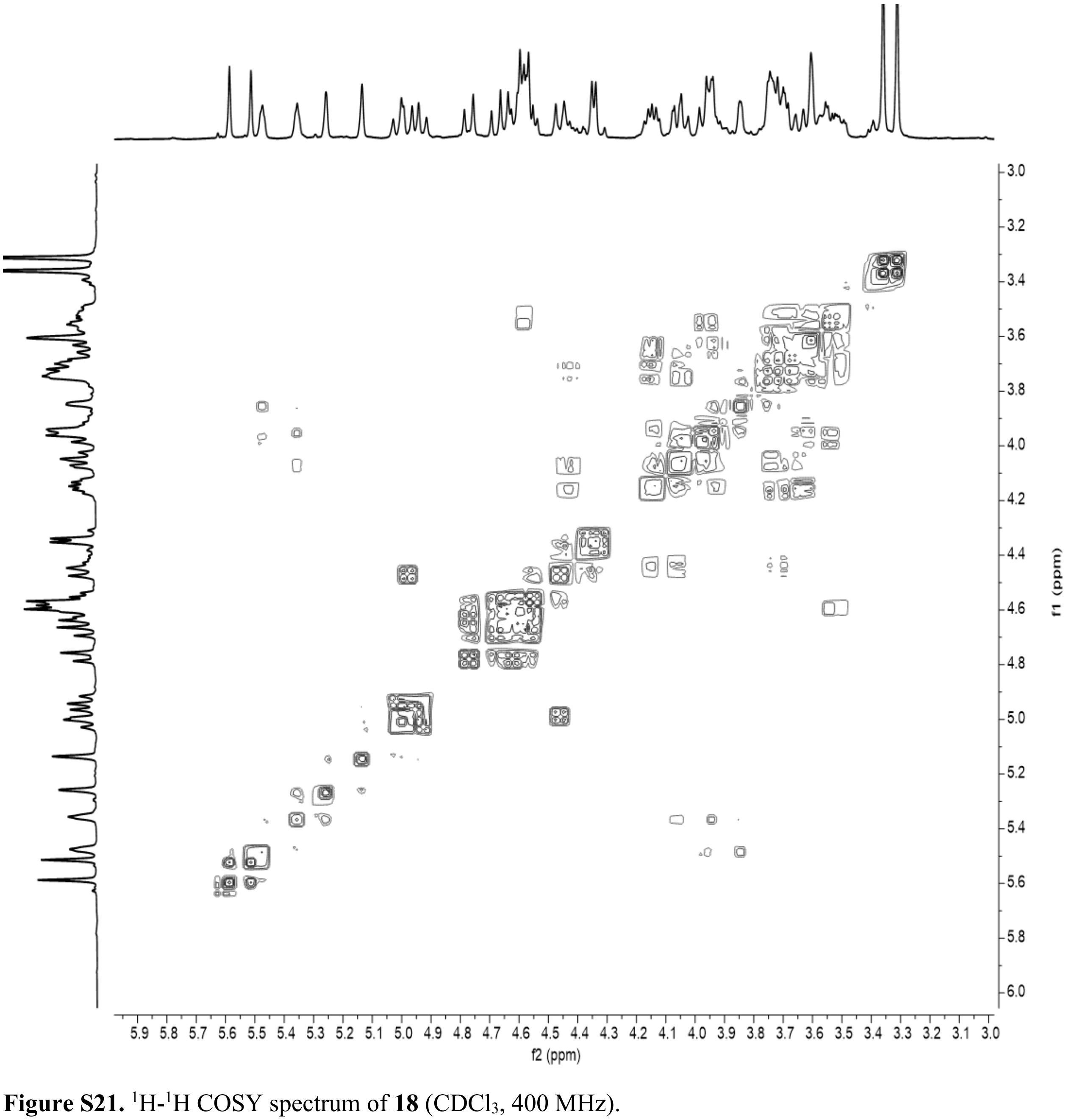

Figure S21. ^{1}H-^{1}H COSY spectrum of **18** ($CDCl_3$, 400 MHz).

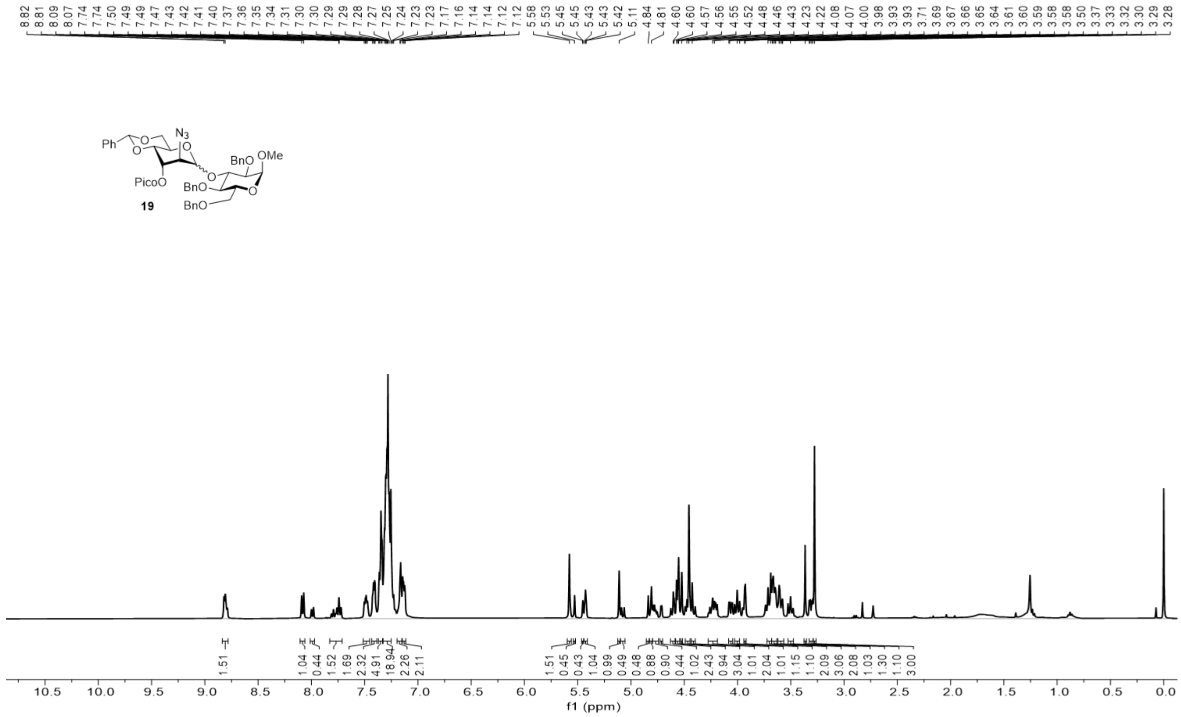

Figure S22. ^{1}H NMR spectrum of **19** ($CDCl_3$, 400 MHz).

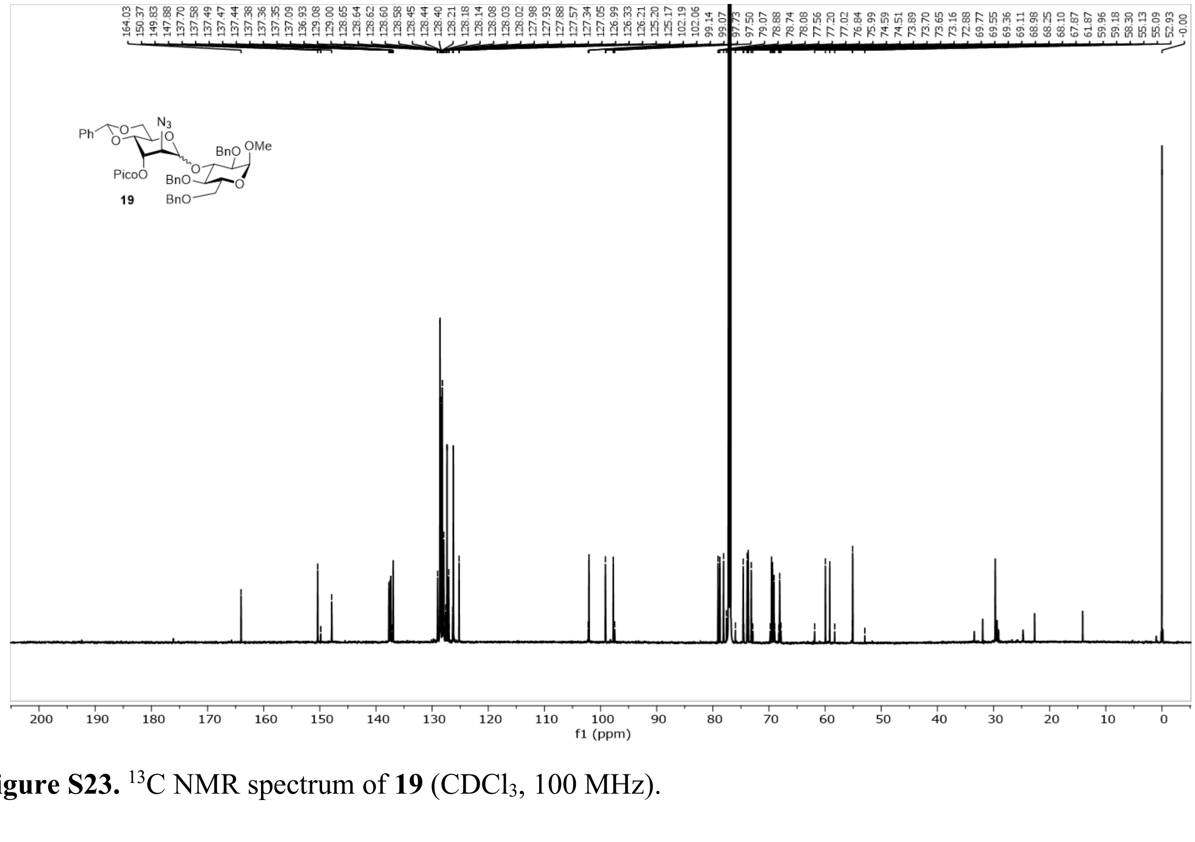

Figure S23. ^{13}C NMR spectrum of **19** ($CDCl_3$, 100 MHz).

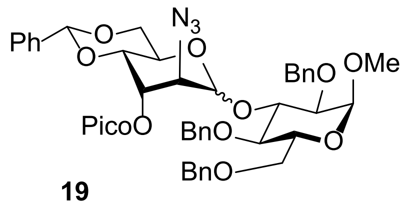

19

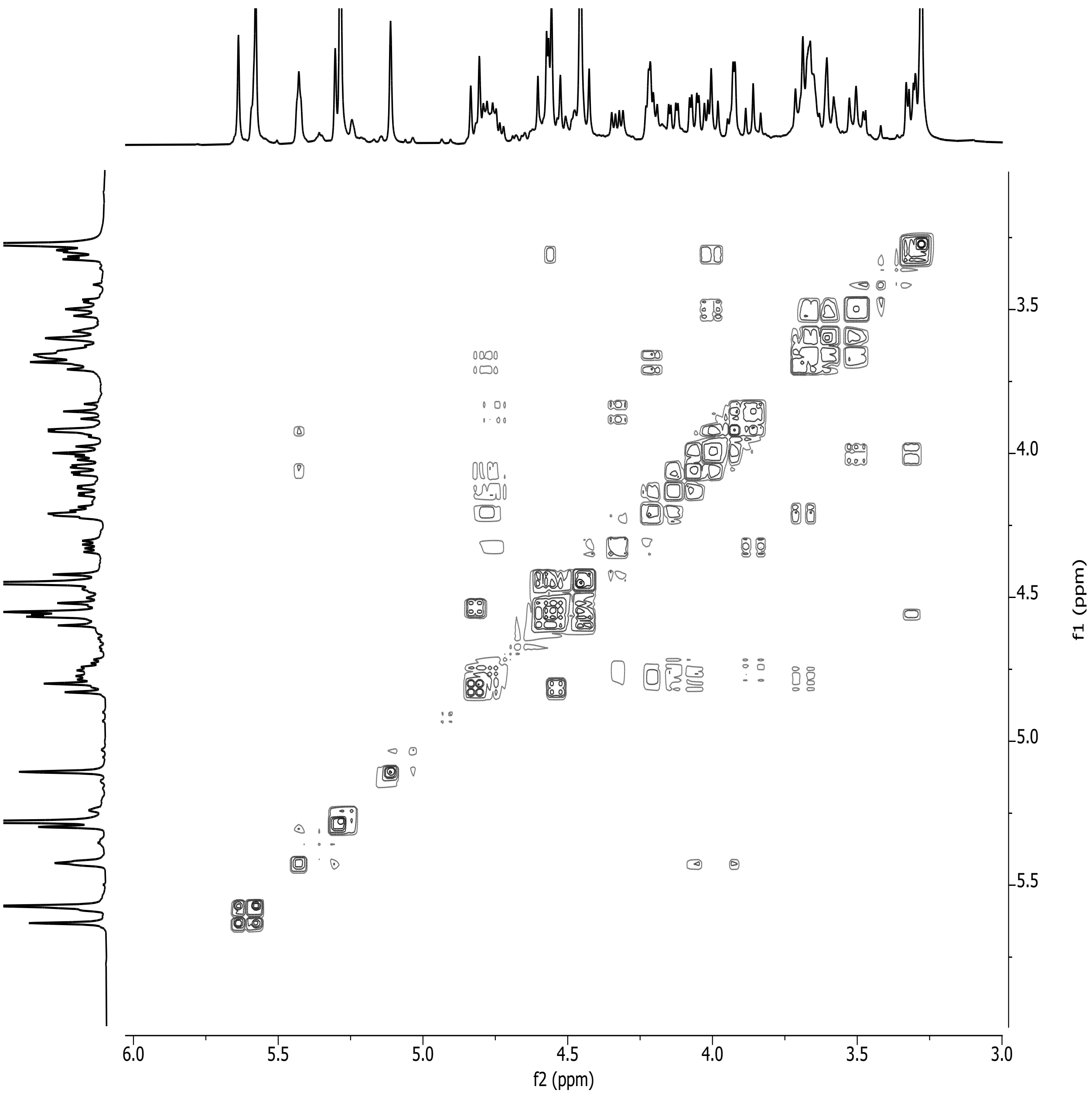

Figure S24. ^{1}H-^{1}H COSY spectrum of **19** ($CDCl_3$, 400 MHz).

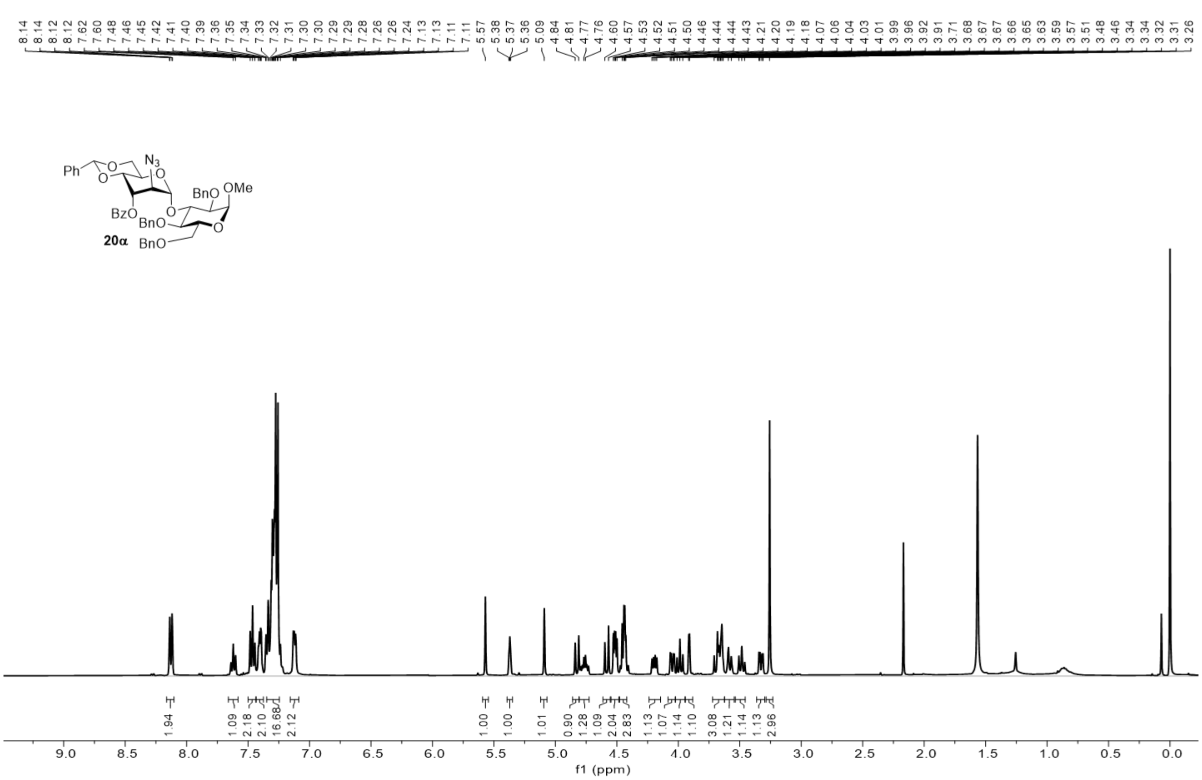

Figure S25. ^{1}H NMR spectrum of **20α** ($CDCl_3$, 400 MHz).

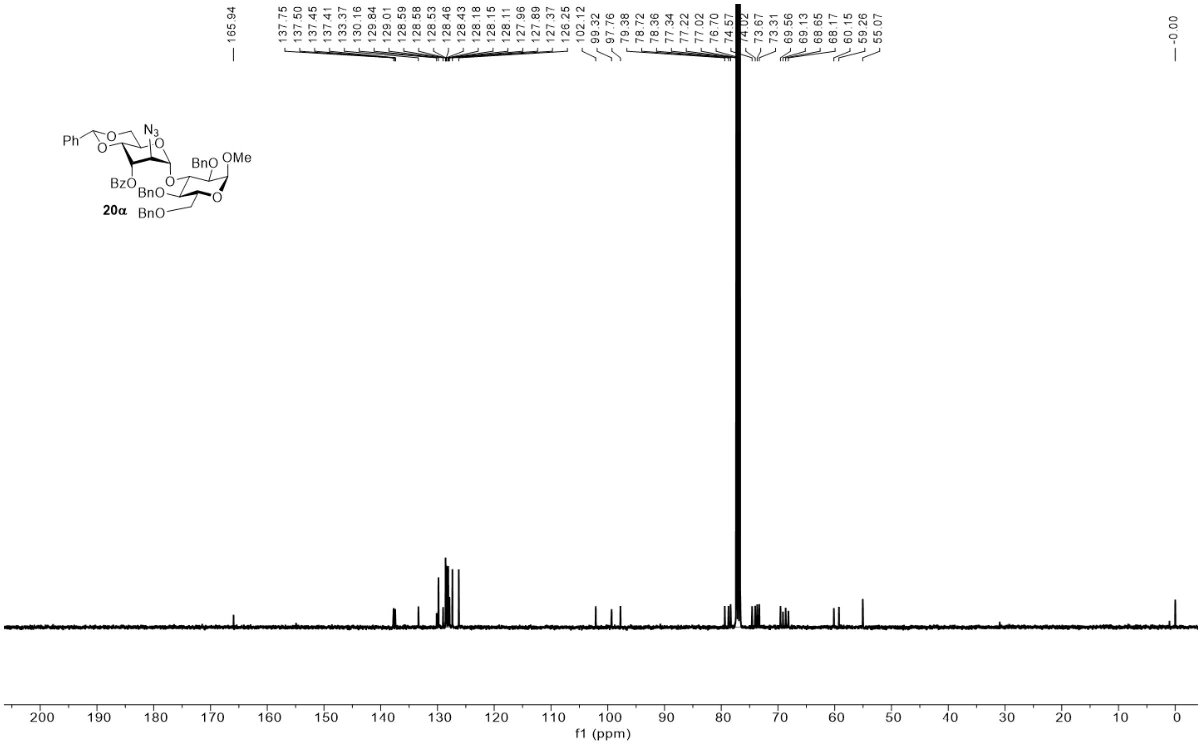

Figure S26. ^{13}C NMR spectrum of **20α** ($CDCl_3$, 100 MHz).

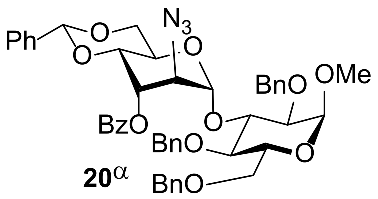

20α

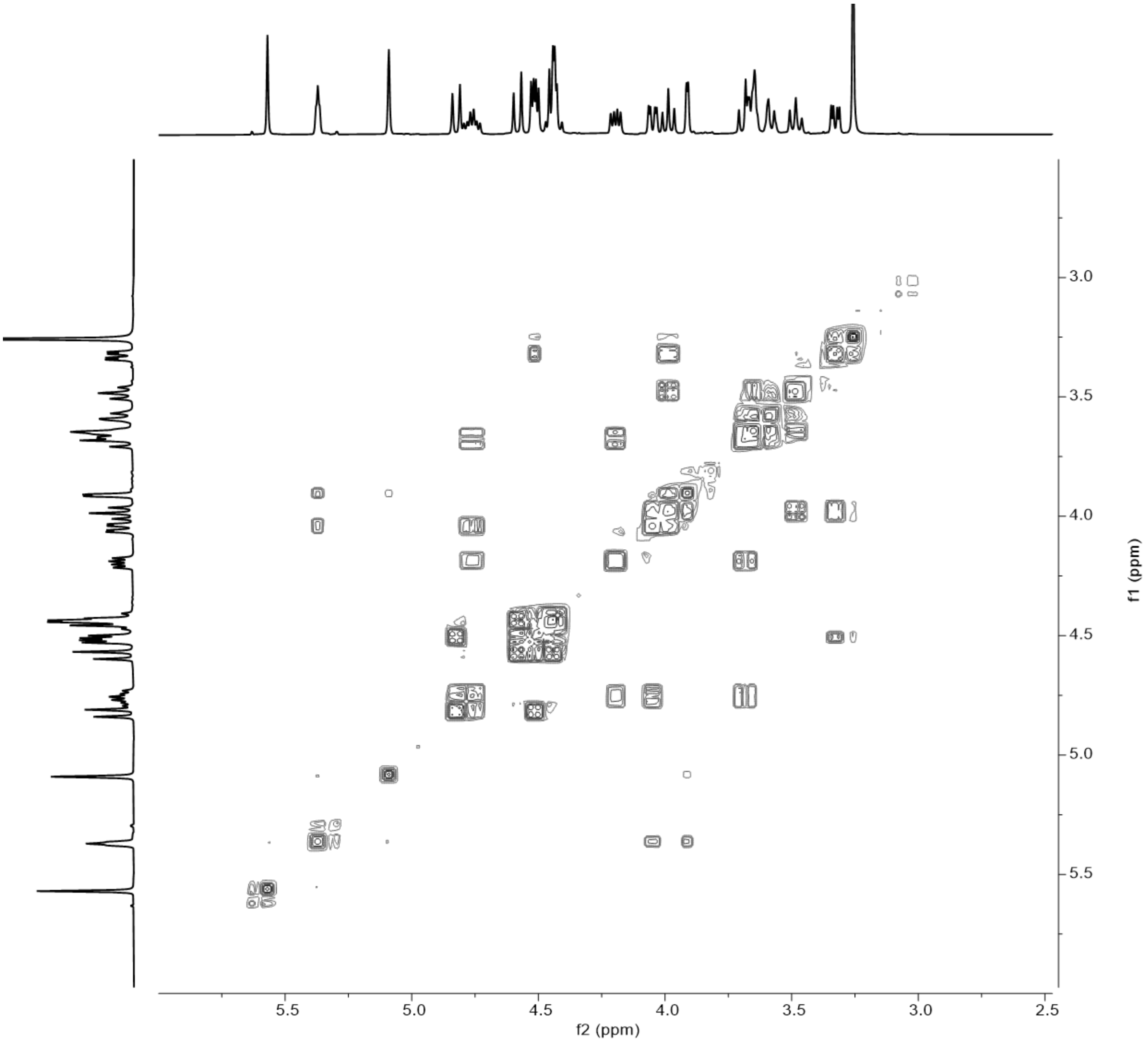

Figure S27. ¹H-¹H COSY spectrum of **20** ($CDCl_3$, 400 MHz).

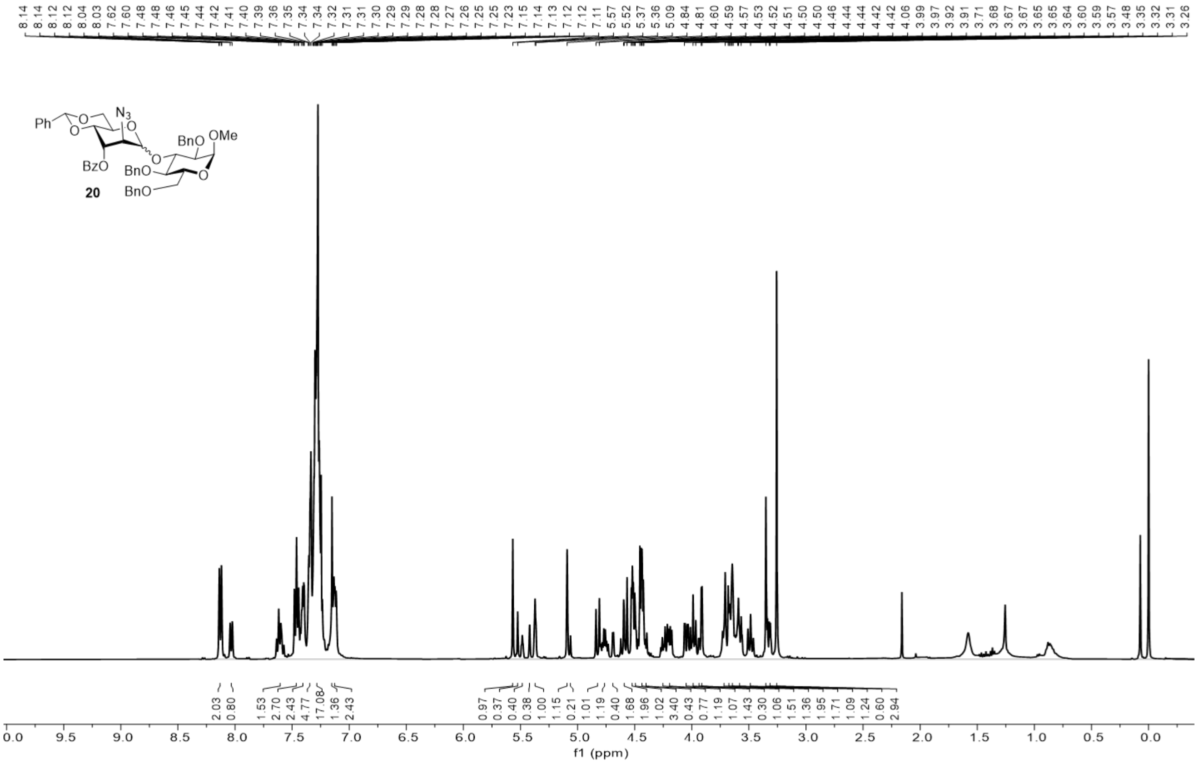

Figure S28. ^{1}H NMR spectrum of **21** ($CDCl_3$, 400 MHz).

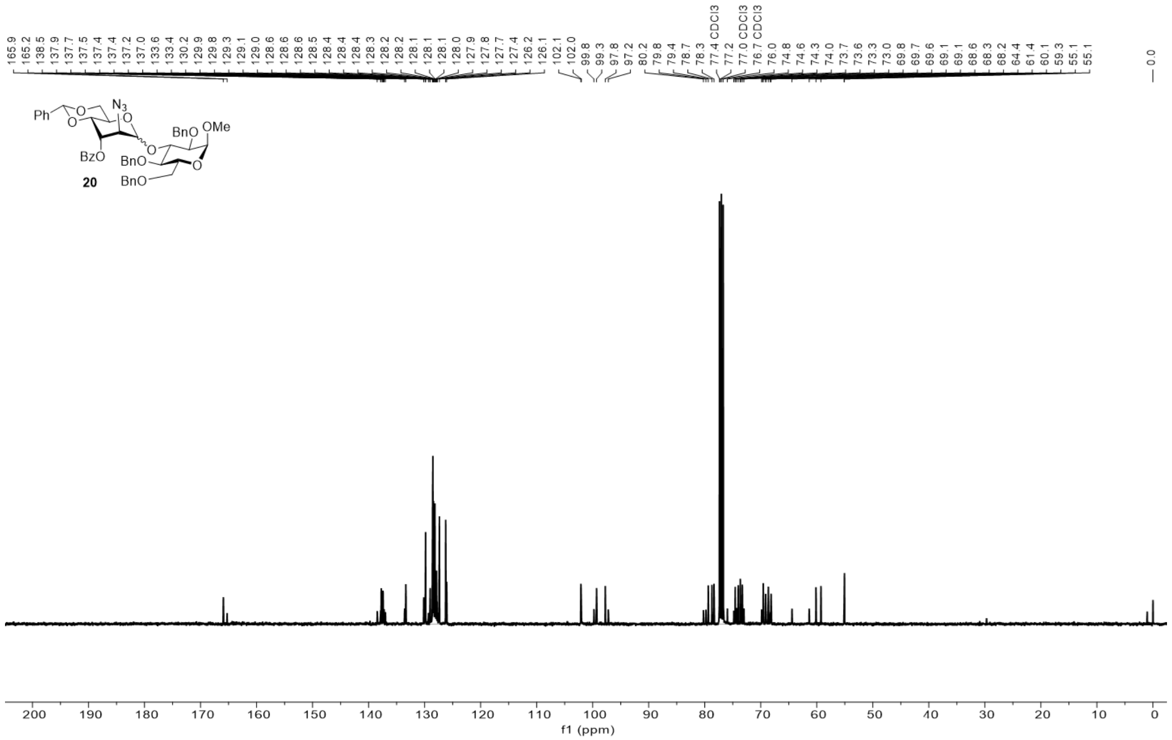

Figure S29. ^{13}C NMR spectrum of **21** ($CDCl_3$, 100 MHz).

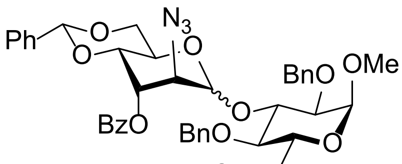

20

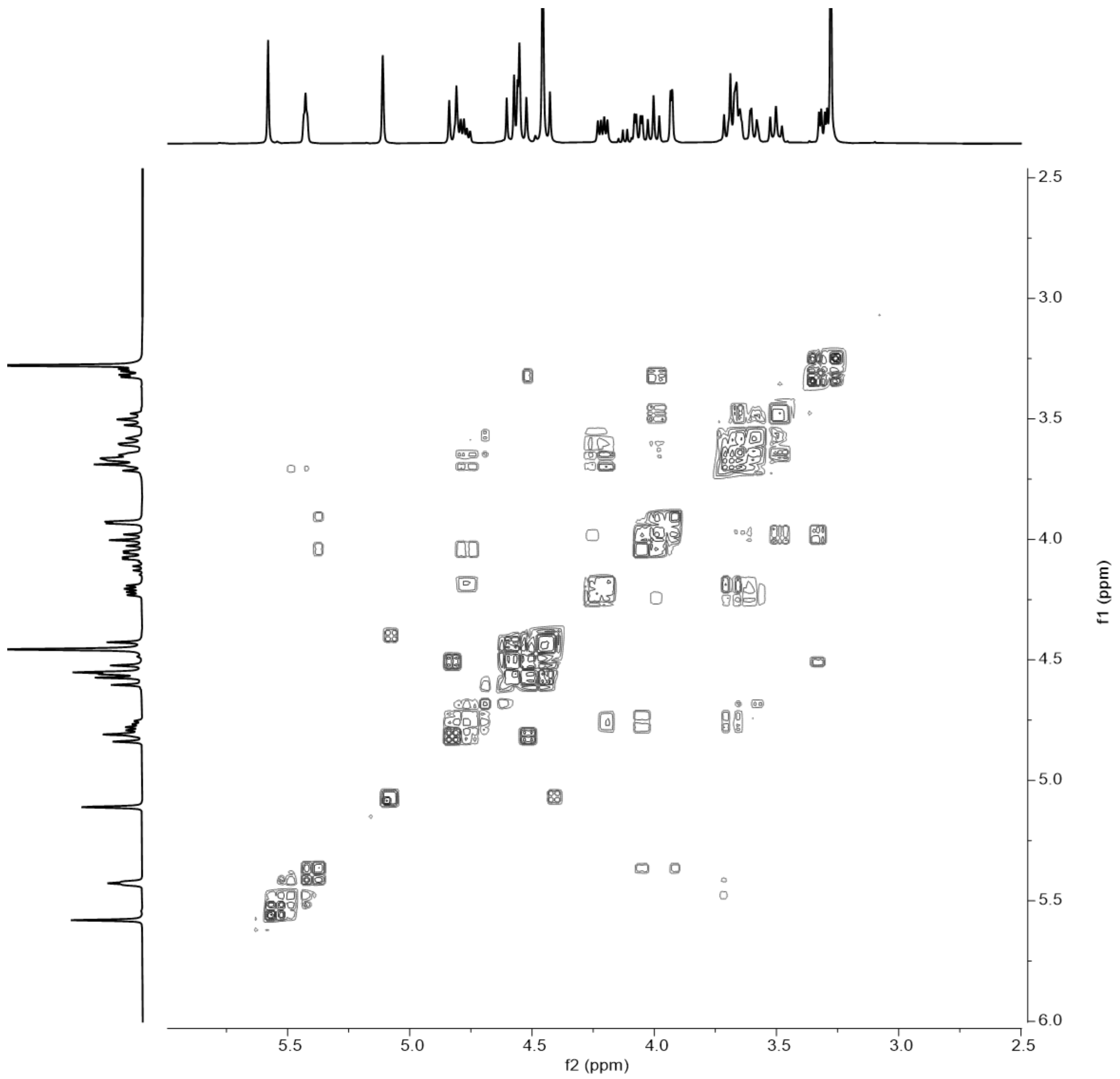

Figure S30. ^{1}H-^{1}H COSY spectrum of **21** ($CDCl_3$, 400 MHz).

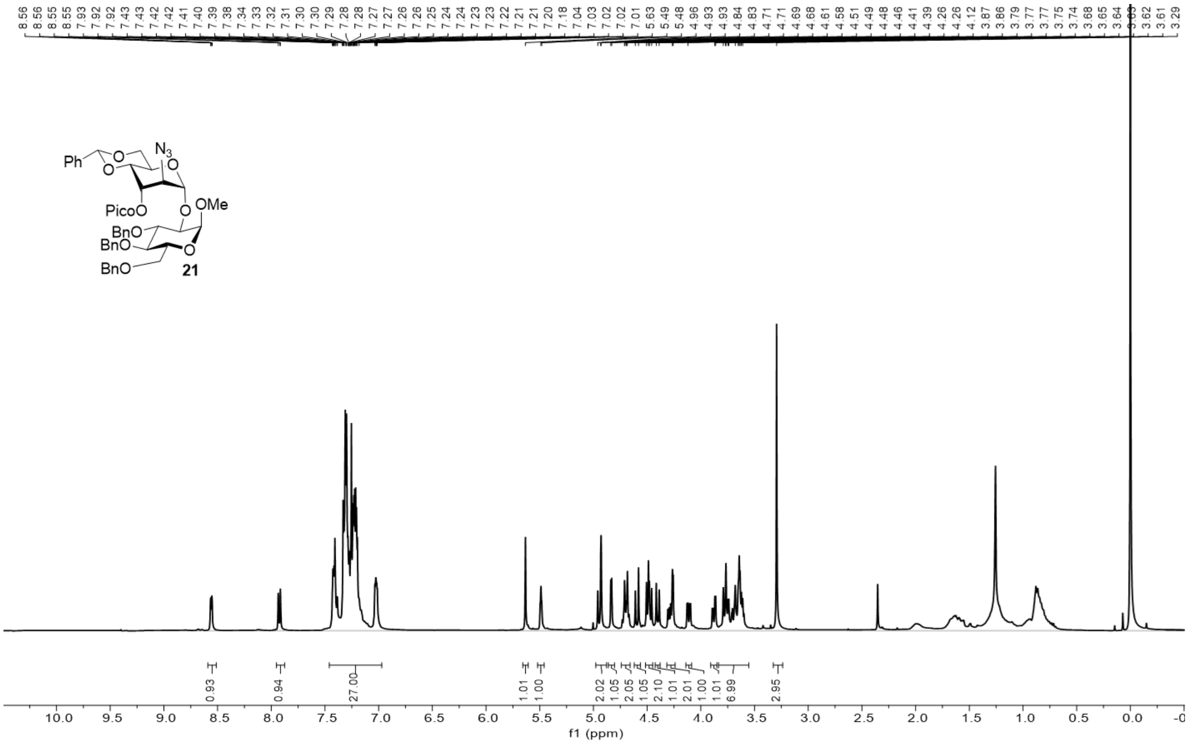

Figure S31. ^{1}H NMR spectrum of **21** ($CDCl_3$, 400 MHz).

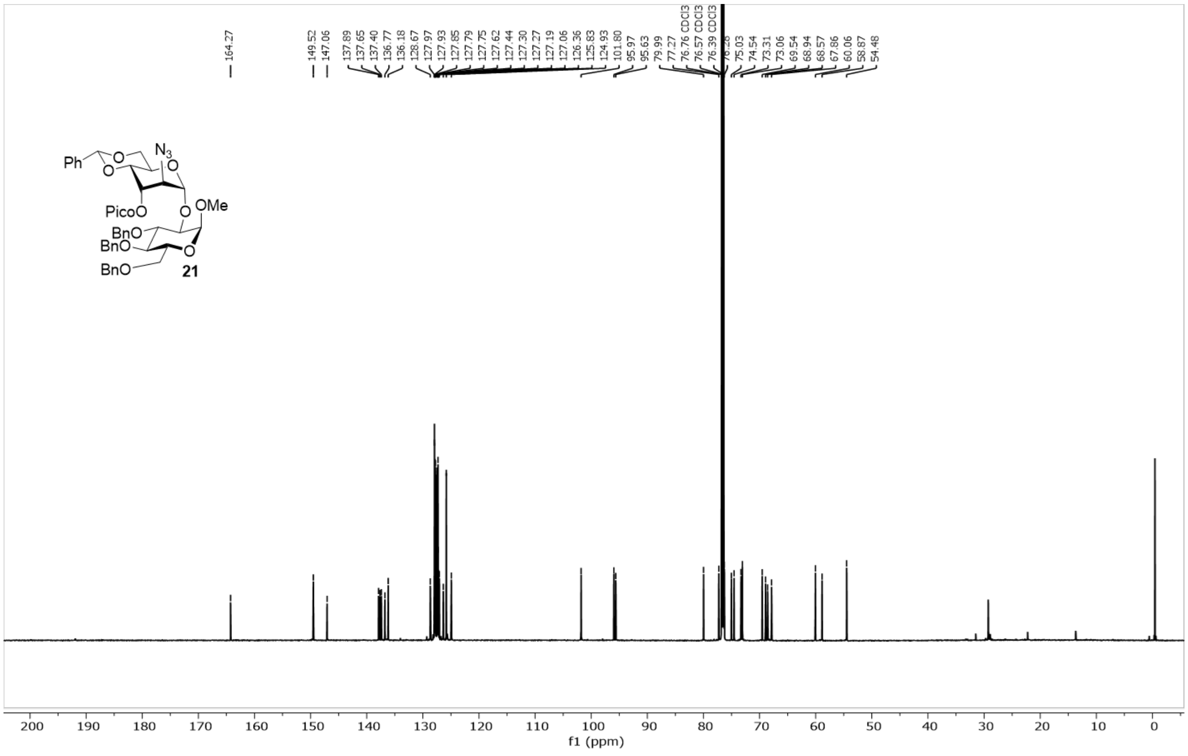

Figure S32. ^{13}C NMR spectrum of **21** ($CDCl_3$, 100 MHz).

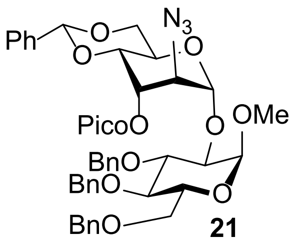

21

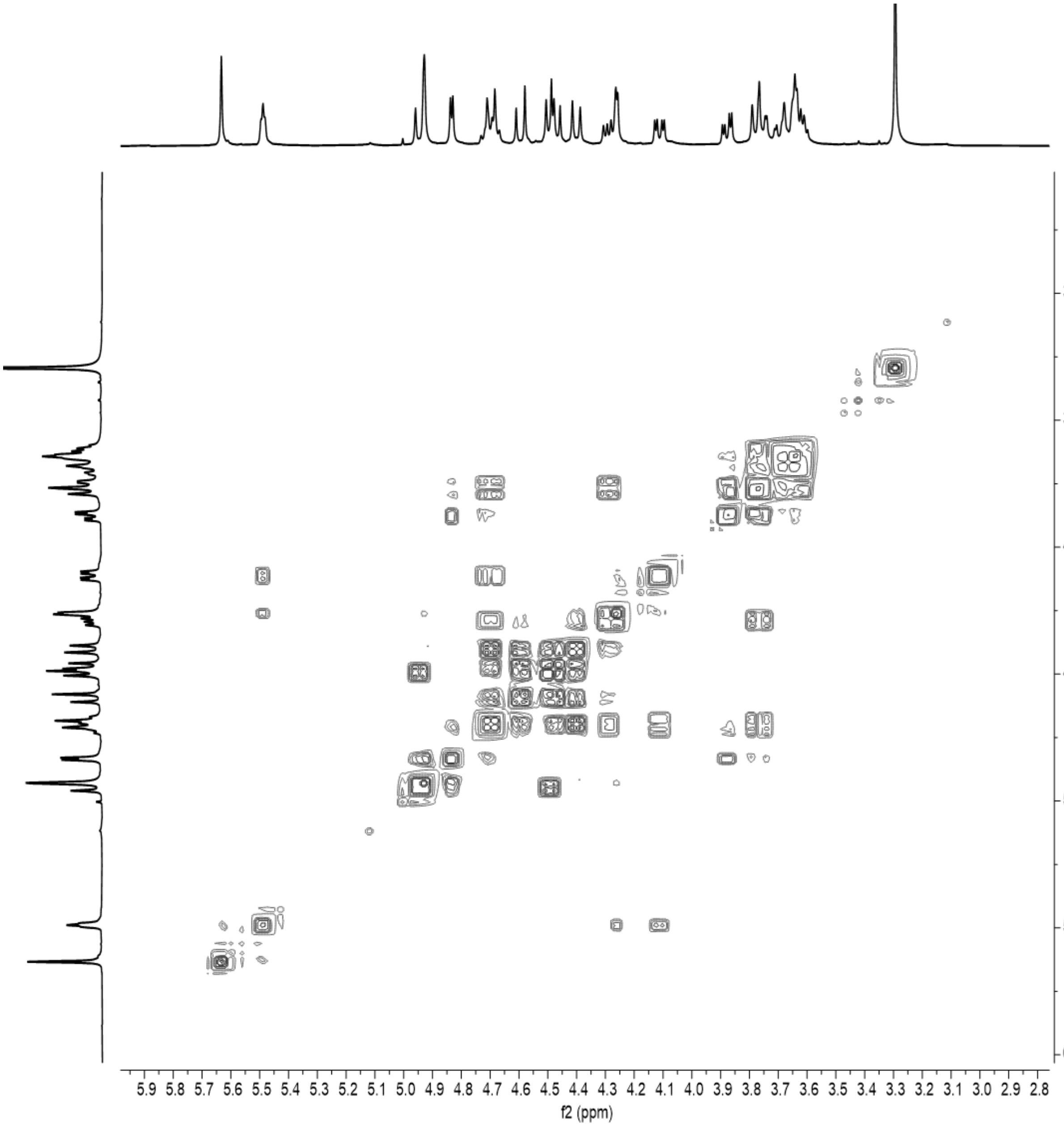

Figure S33. ^1H-^1H COSY spectrum of **21** ($CDCl_3$, 400 MHz).

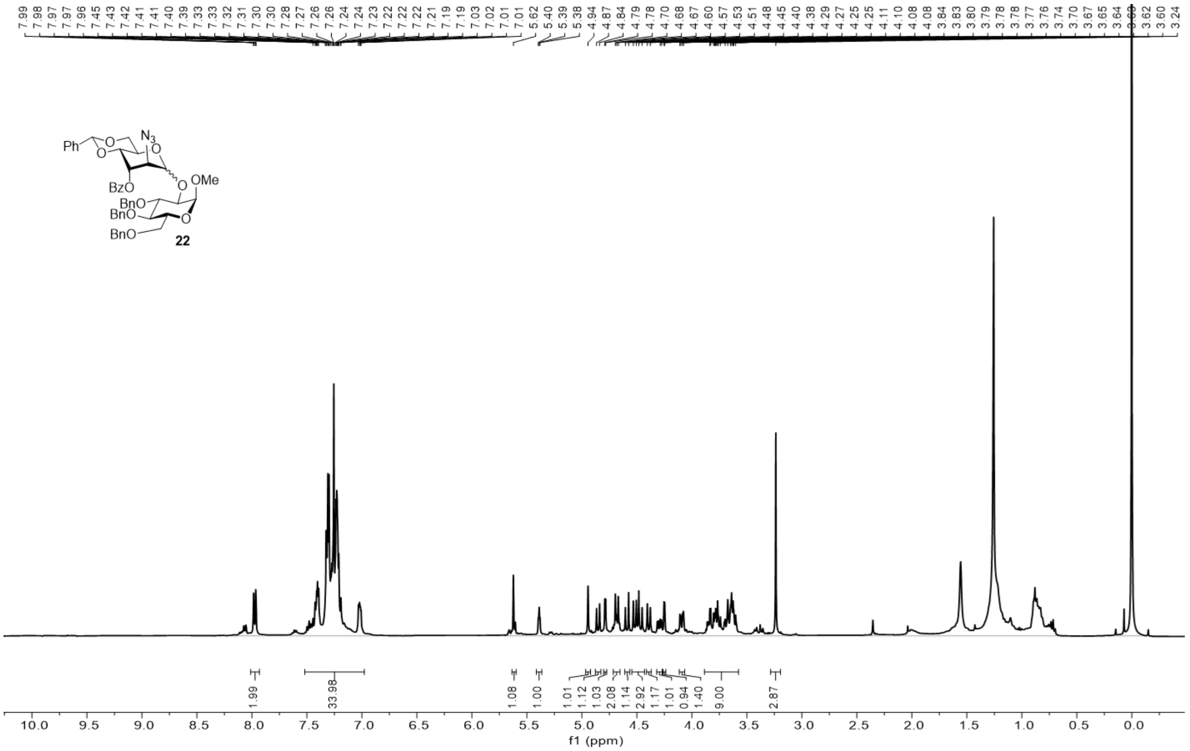

Figure S34. ^{1}H NMR spectrum of **22** ($CDCl_3$, 400 MHz).

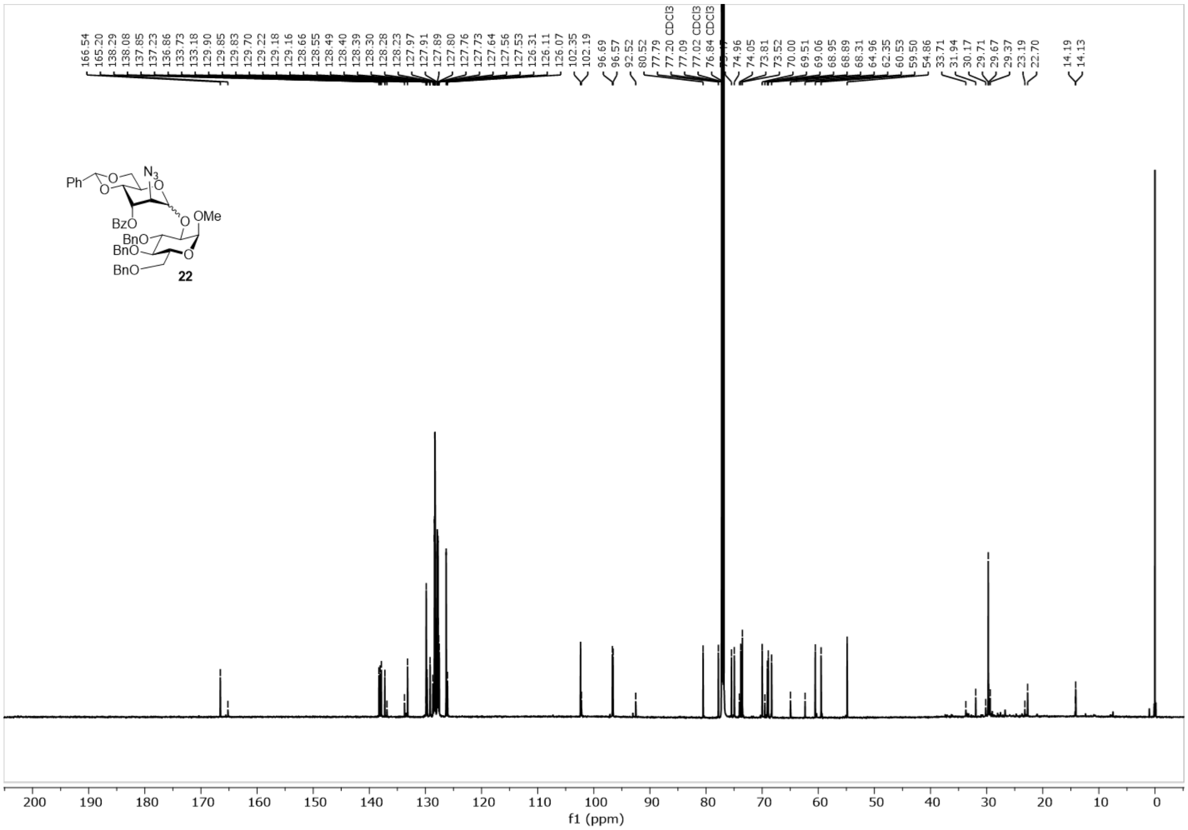

Figure S35. ^{13}C NMR spectrum of **22** ($CDCl_3$, 100 MHz).

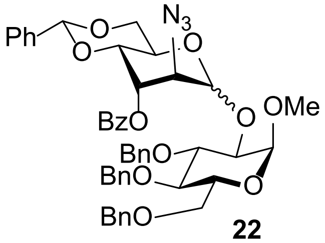

22

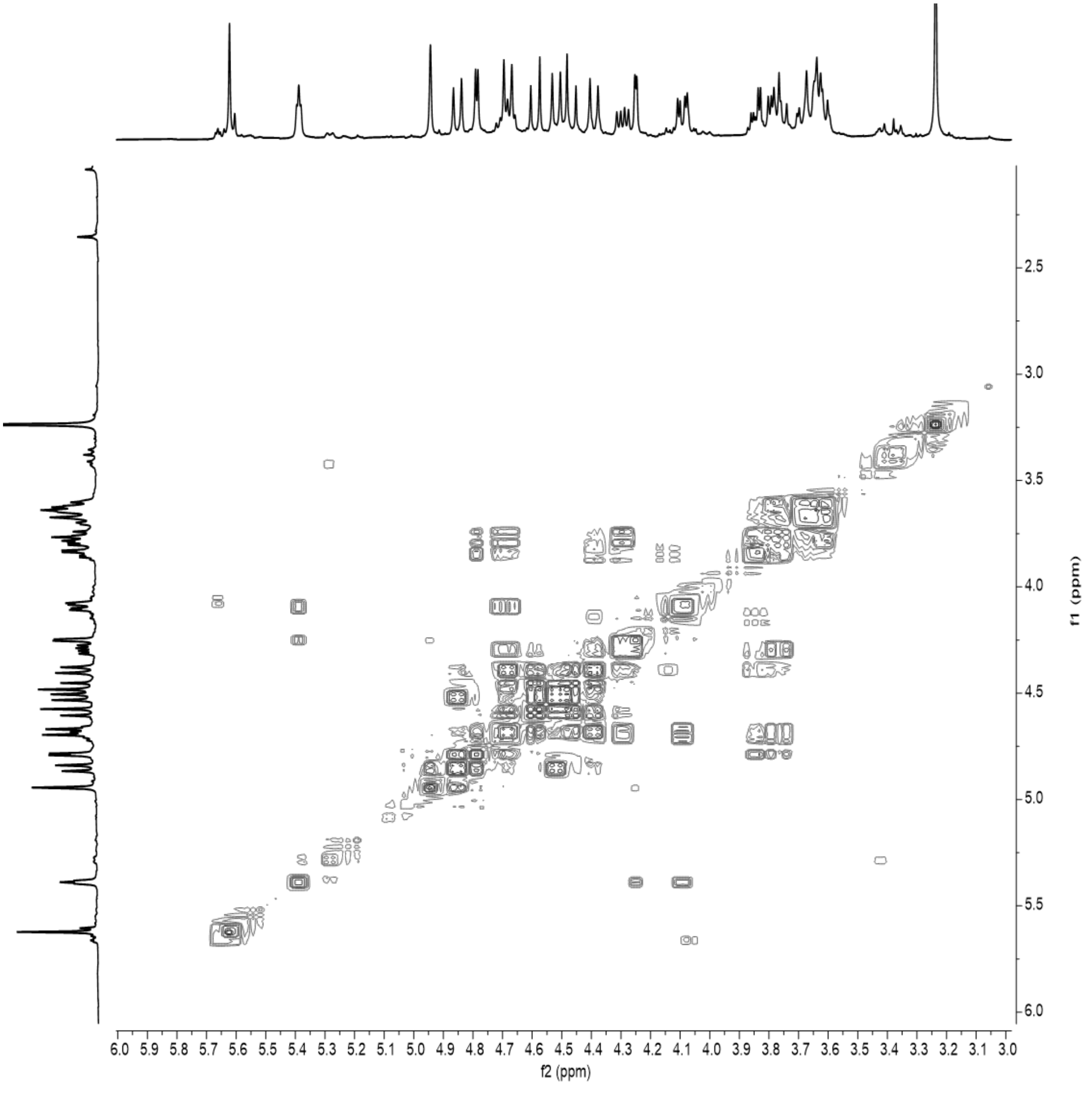

Figure S36. ^{1}H-^{1}H COSY spectrum of **22** ($CDCl_3$, 400 MHz).

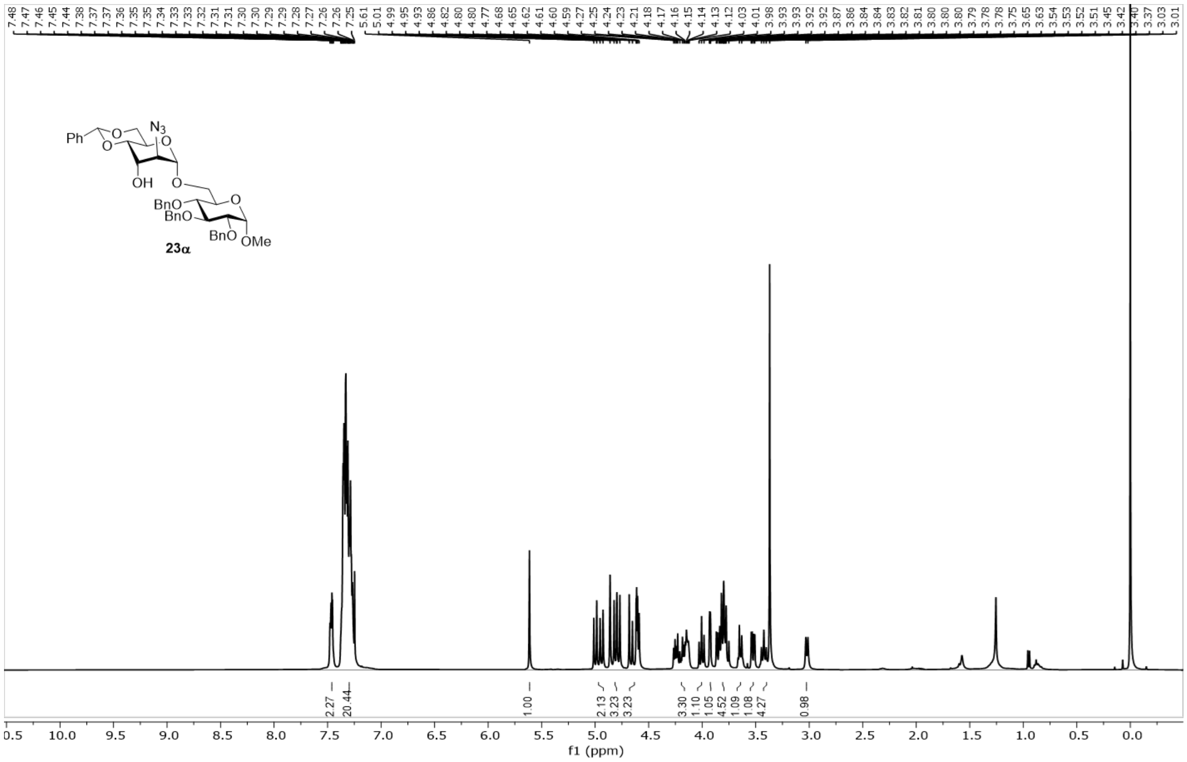

Figure S37. ^{1}H NMR spectrum of **23α** ($CDCl_3$, 400 MHz).

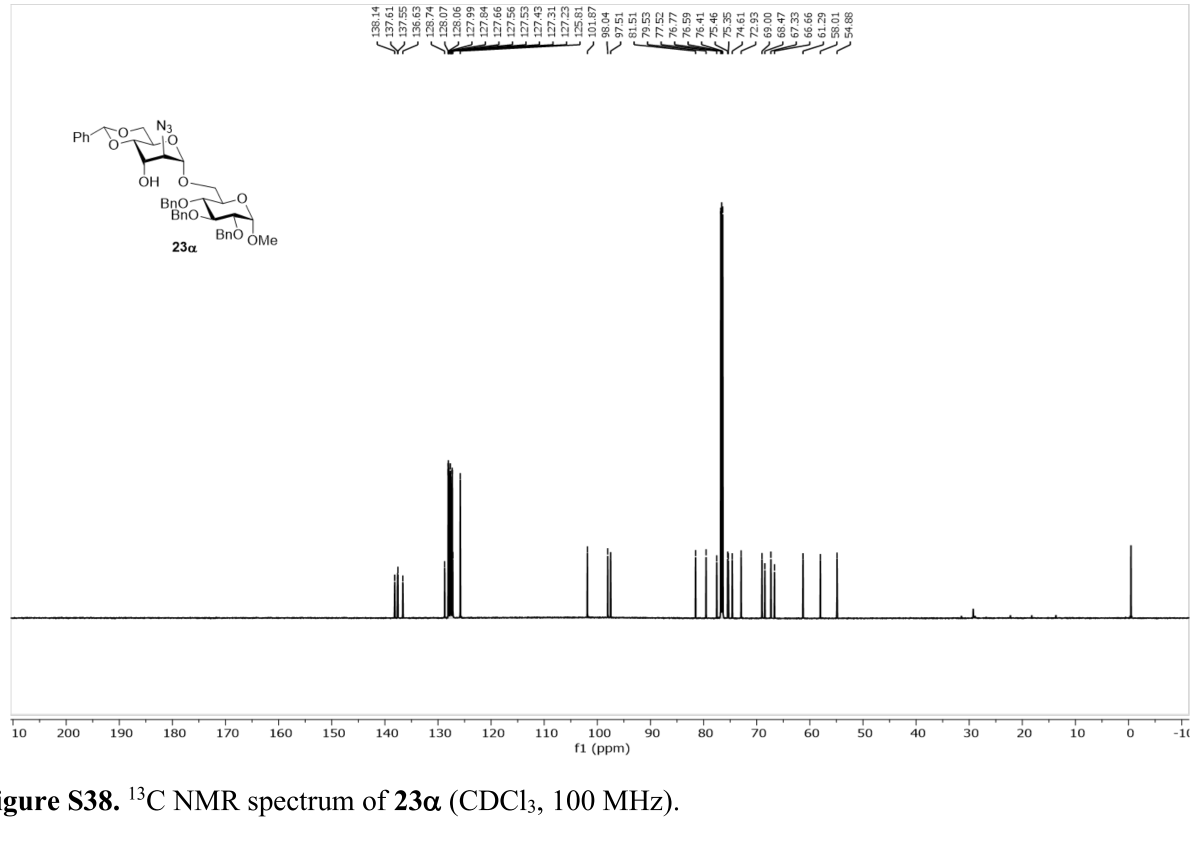

Figure S38. ^{13}C NMR spectrum of **23α** ($CDCl_3$, 100 MHz).

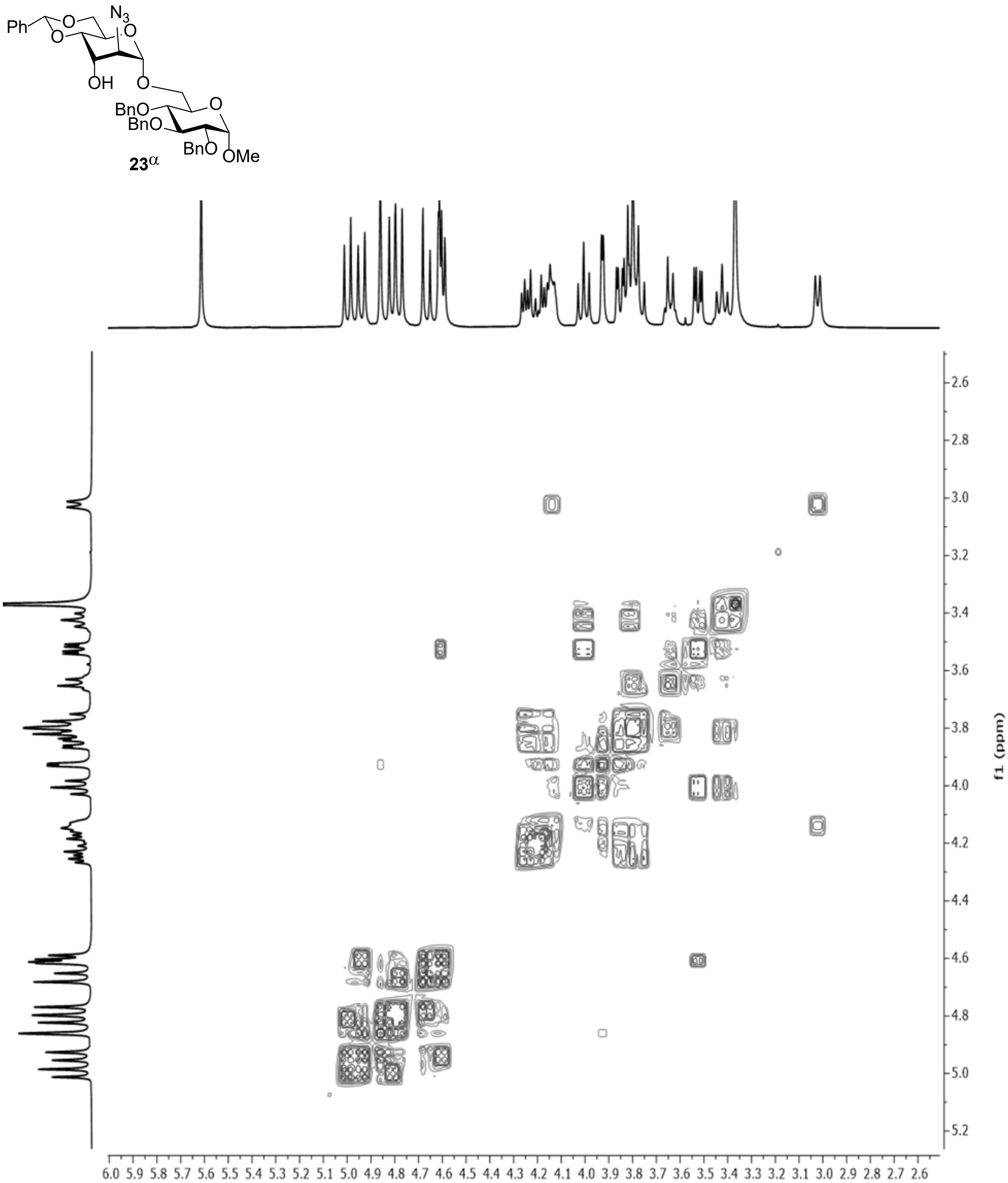

Figure S39 ^{1}H-^{1}H COSY spectrum of **23α** ($CDCl_3$, 400 MHz).

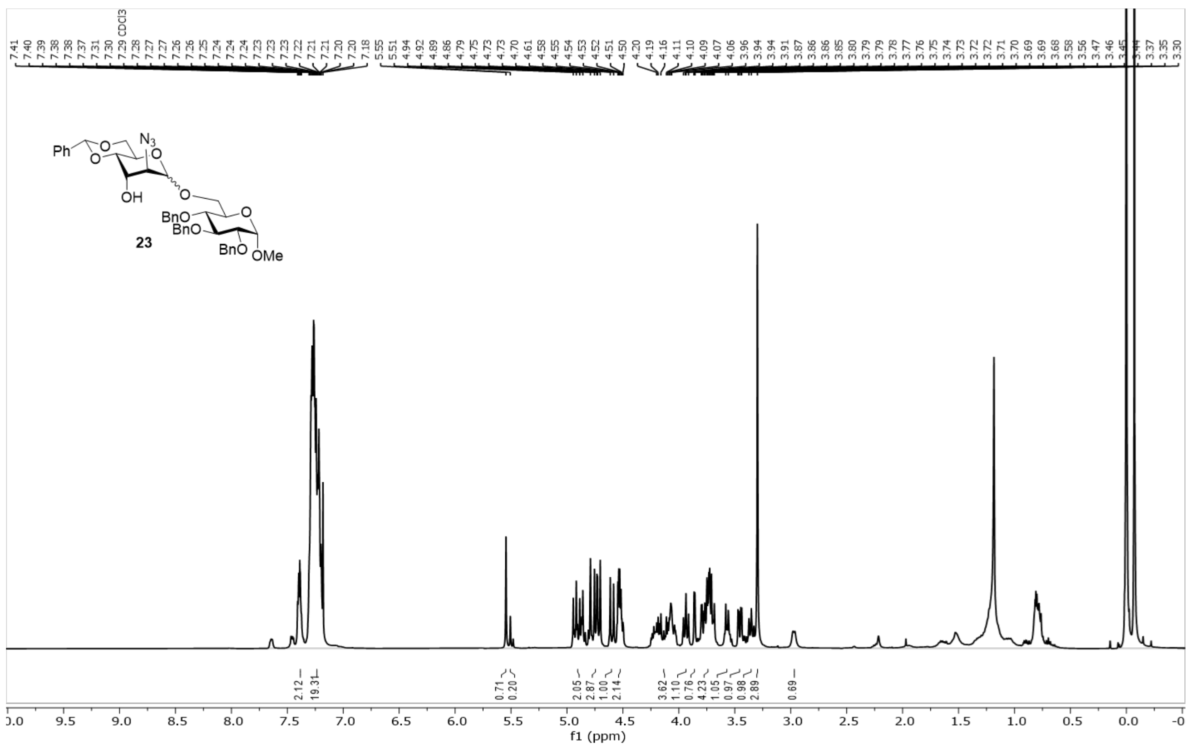

Figure S40. ^{1}H NMR spectrum of **23** ($CDCl_3$, 400 MHz).

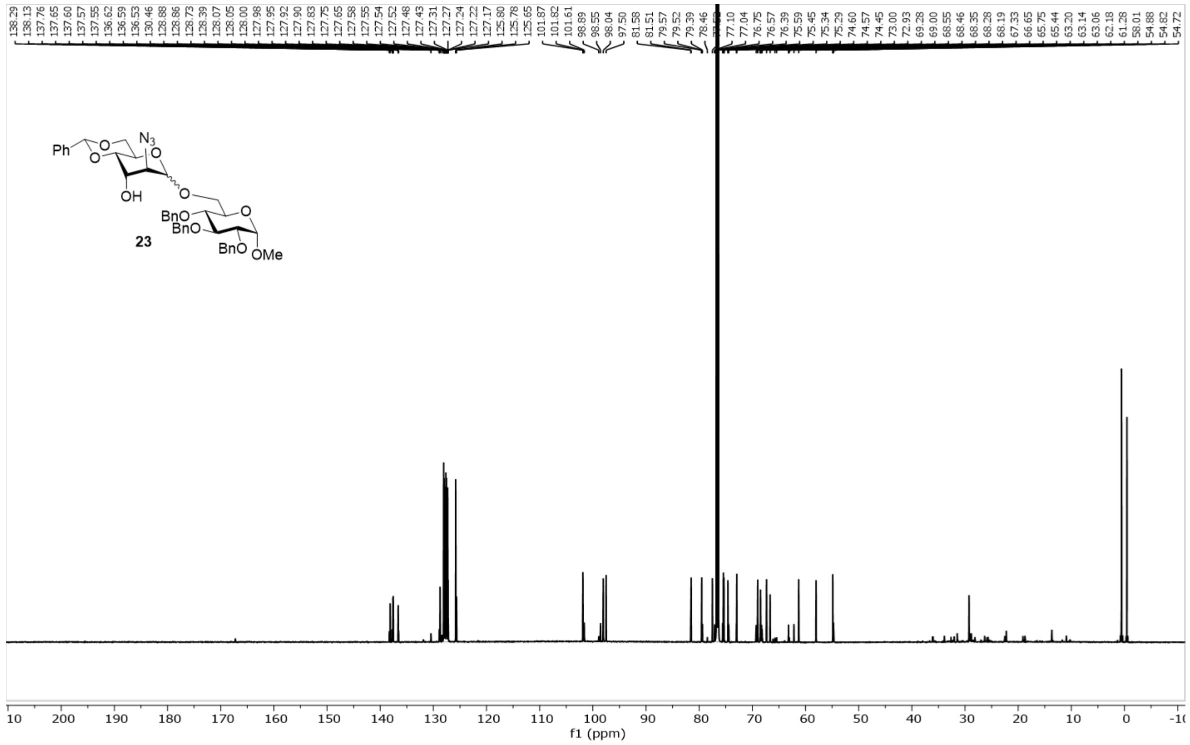

Figure S41. ^{13}C NMR spectrum of **23** ($CDCl_3$, 100 MHz).

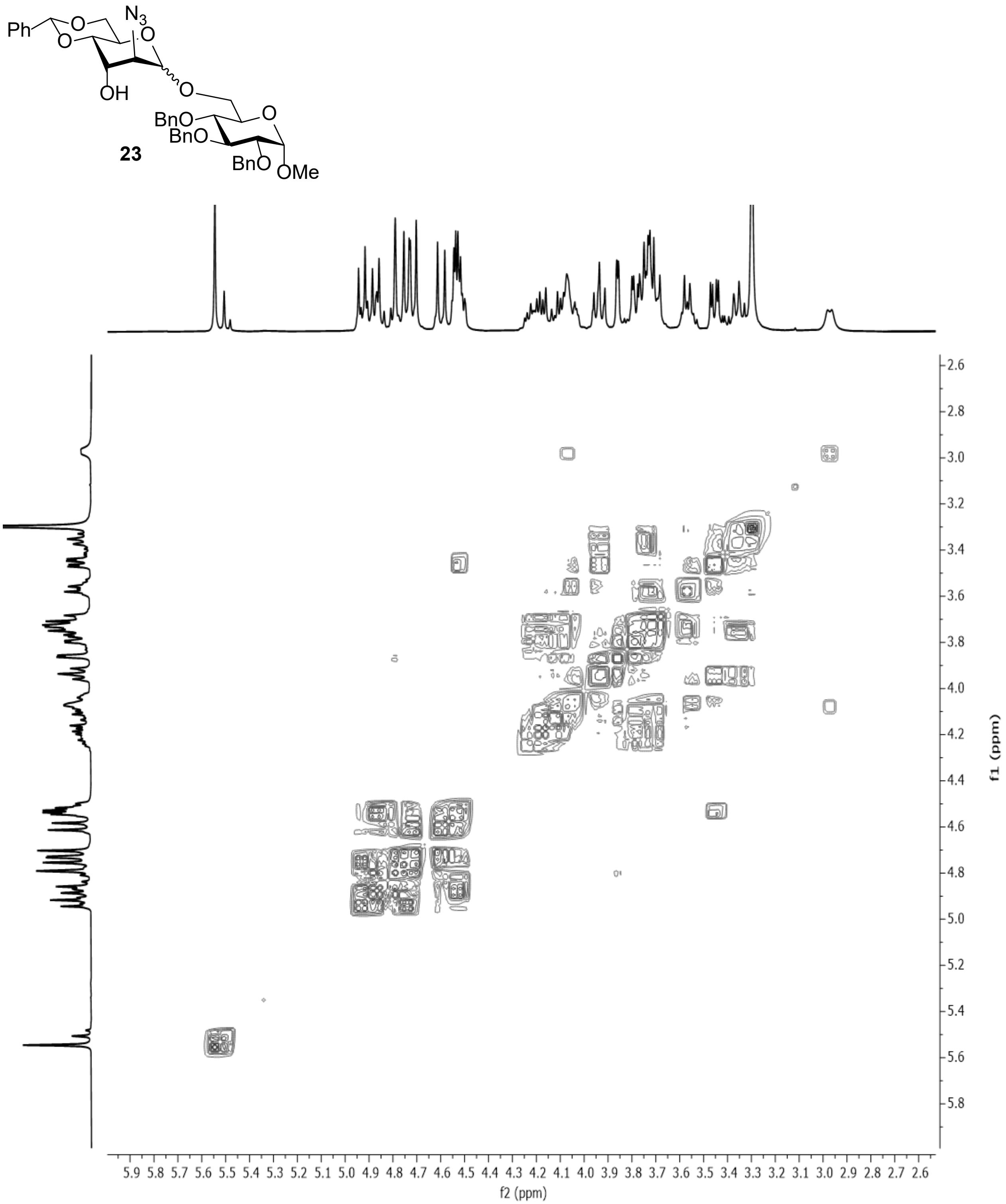

Figure S42 ^{1}H-^{1}H COSY spectrum of **23** ($CDCl_3$, 400 MHz).
